# Curcumin analogue FLLL32 induces ferroptosis by targeting the STAT3-GPX4/SLC40A1 axis in colorectal cancer

**DOI:** 10.1186/s43556-026-00575-5

**Published:** 2026-10-20

**Authors:** Tong Wu, Mengqiu Hao, Qiuye Luo, Sicheng Liu, Yaguang Zhang, Yang Zhang, Xuyang Yang, Junhong Han

**Affiliations:** 1 Laboratory of Gastrointestinal Tumor Epigenetics and Genomics, State Key Laboratory of Biotherapy and Cancer Center, Frontiers Science Center for Disease-related Molecular Network, West China Hospital, Sichuan University, Chengdu, 610041 China; 2 Colorectal Cancer Center, West China Hospital, Sichuan University, Chengdu, China

**Keywords:** CRC, FLLL32, Ferroptosis, STAT3 phosphorylation, GPX4, SLC40A1

## Abstract

FLLL32 is a curcumin analogue with reported anti-tumor activity, mainly through inhibition of signal transducer and activator of transcription 3 (STAT3) signaling and induction of apoptosis. However, whether FLLL32 regulates ferroptosis in colorectal cancer (CRC) and the underlying mechanism remain unclear. Here, using a patient-derived CRC organoid screening platform, we identified FLLL32 as a potent anti-CRC compound. FLLL32 suppressed CRC organoid growth, inhibited CRC cell proliferation and migration in vitro, and inhibited xenograft tumor growth in vivo. In parallel, FLLL32 induced ferroptosis-associated iron accumulation, reactive oxygen species (ROS) production, lipid peroxidation, mitochondrial morphological and functional abnormalities. The ferroptosis inhibitor Ferrostatin-1 markedly restored FLLL32-impaired cell viability and reduced ROS, whereas the apoptosis inhibitor Z-VAD-FMK showed weaker protection. These findings suggest that ferroptosis is a major contributor to FLLL32-induced cell death, although apoptotic features are also present. Mechanistically, FLLL32 interacted with STAT3, and Glu638 and Met726 contributed to the FLLL32-STAT3 interaction. FLLL32 also suppressed STAT3 Tyr705 phosphorylation and nuclear signaling, thereby disrupting pSTAT3-mediated transcriptional regulation of glutathione peroxidase 4 (GPX4) and solute carrier family 40 member 1 (SLC40A1), weakening lipid peroxide detoxification and cellular iron efflux, promoting intracellular iron accumulation, and enhancing ferroptosis in CRC cells. These findings define a STAT3-GPX4/SLC40A1-dependent mechanism underlying the anti-CRC activity of FLLL32 and support further investigation of STAT3-associated ferroptosis regulation as a potential therapeutic vulnerability in CRC.

**Supplementary Information:**

The online version contains supplementary material available at https://doi.org/10.1186/s43556-026-00575-5.

## Introduction

CRC is one of the most common malignancies and a major cause of cancer-related mortality worldwide, accounting for approximately 10% of all cancer cases [1]. Although surgery, chemotherapy, targeted therapy, and immunotherapy have improved the clinical management of CRC, treatment resistance, tumor recurrence, and adverse reactions continue to limit therapeutic efficacy, especially in patients with advanced or metastatic disease [2–6]. Therefore, identifying effective and low-toxicity therapeutic agents remains an important goal for CRC treatment.

Patient-derived organoids (PDOs) retain key histological, genetic, and drug-response features of the original tumors and provide a clinically relevant platform for functional drug screening. Compared with conventional cell lines, PDO models better preserve inter-patient heterogeneity and may improve the translational value of drug discovery [7–11]. In response to the limitations of conventional therapies, there is growing interest in developing natural-product-derived compounds as potential anticancer agents. One promising avenue is the use of natural compounds and their structurally optimized derivatives with anticancer properties. Curcumin, derived from turmeric, has shown significant anticancer activity, but its clinical utility is limited by poor solubility, low bioavailability, and limited pharmacological stability [12]. FLLL32 is a structurally modified curcumin analogue developed as a STAT3-targeting inhibitor with improved binding potency and selectivity. Previous studies have shown that FLLL32 suppresses STAT3 phosphorylation and DNA-binding activity, thereby inhibiting STAT3-dependent transcription and inducing apoptosis in several tumor models [13, 14].

Ferroptosis is a regulated form of cell death triggered by iron-dependent oxidation. It is distinct from apoptosis and is characterized by excessive accumulation of lipid peroxides, disruption of iron homeostasis, and impairment of antioxidant defense systems [15, 16]. Increasing evidence indicates that ferroptosis contributes to CRC progression and treatment response, and modulation of ferroptosis has emerged as an important direction in CRC research [17, 18]. Key regulators of ferroptosis include GPX4, SLC7A11, and iron transport proteins [19, 20]. Among these, GPX4 protects cells from ferroptosis by reducing phospholipid hydroperoxides [21], whereas SLC40A1, also known as ferroportin, mediates cellular iron export and limits intracellular iron accumulation. Thus, coordinated disruption of lipid peroxide detoxification and iron efflux may increase ferroptotic stress in tumor cells.

STAT3 is a transcription factor frequently activated in CRC and contributes to tumor proliferation, survival, migration, immune escape, and therapeutic resistance. Upon phosphorylation at Tyr705, STAT3 undergoes dimerization and nuclear translocation, where it binds to promoter regions and regulates target gene transcription [22]. Recent studies have linked STAT3 signaling to ferroptosis regulation, especially through transcriptional control of GPX4 [23–25]. However, whether STAT3 coordinates both lipid peroxide detoxification and iron efflux programs in CRC remains unclear.

In this study, we used a CRC PDO-based screening platform to identify FLLL32 as an active compound against CRC models. We found that FLLL32 suppressed CRC organoid growth, inhibited CRC cell proliferation and migration, and induced ferroptosis-related lipid peroxidation, iron accumulation, and mitochondrial dysfunction. Mechanistically, FLLL32 reduced STAT3 phosphorylation and nuclear pSTAT3 levels, decreased pSTAT3 occupancy at the GPX4 and SLC40A1 promoters, and suppressed GPX4/SLC40A1 transcription. Functional rescue experiments further showed that restoration of the STAT3-GPX4/SLC40A1 axis attenuated FLLL32-induced ferroptotic stress. These findings reveal a mechanism by which FLLL32 promotes ferroptotic stress in CRC cells through inhibition of the STAT3-GPX4/SLC40A1 axis and suggest that targeting STAT3-dependent ferroptosis-defense programs may offer a potential direction for ferroptosis-based therapeutic development in CRC.

## Results

### Patient-derived CRC organoid-based screening identifies FLLL32 as a candidate anti-CRC compound

**Fig. 1:**
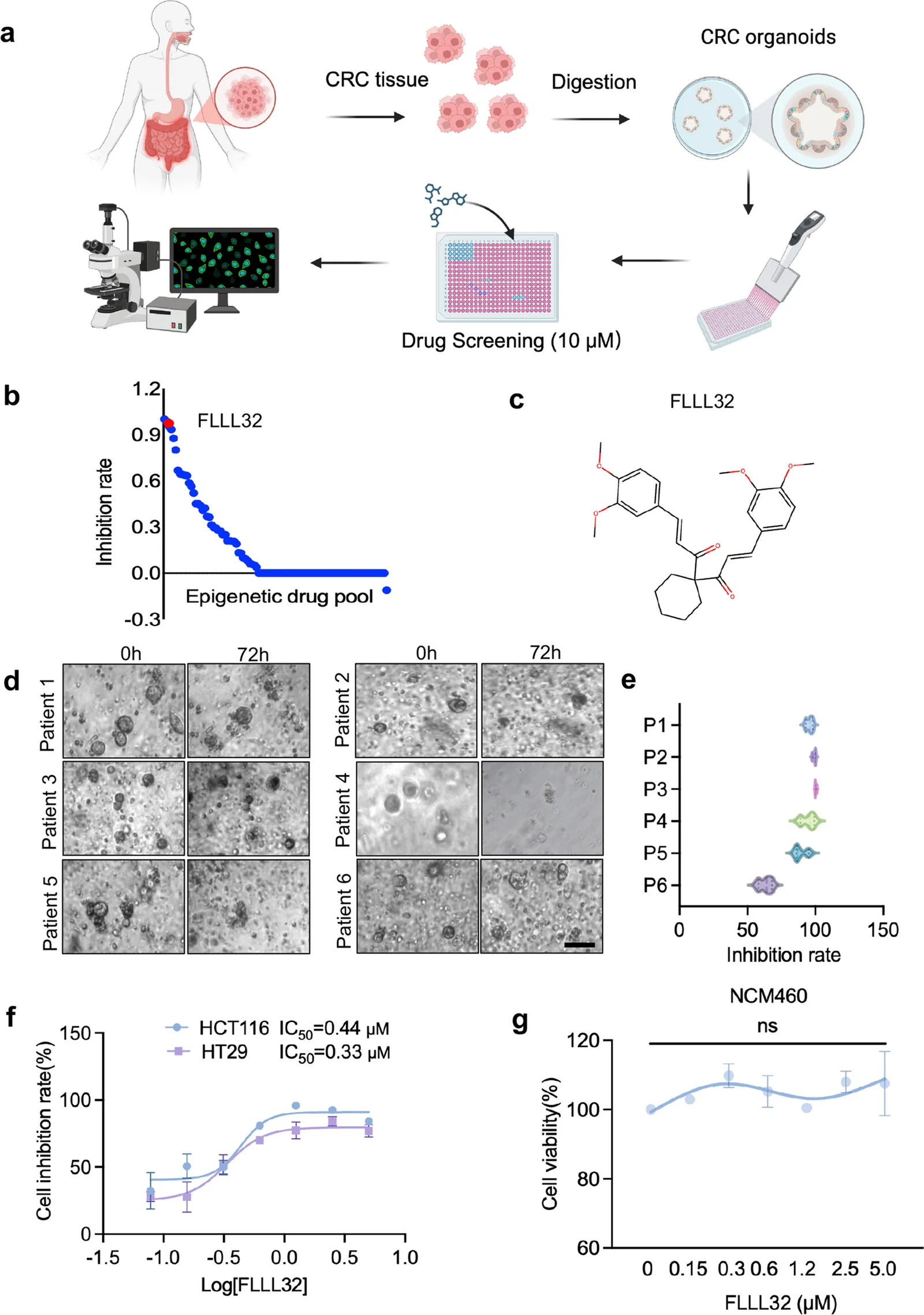
Patient-derived CRC organoid-based screening identifies FLLL32 as a candidate anti-CRC compound. **a** Schematic workflow of patient-derived colorectal cancer organoid establishment and compound screening in a 384-well plate. **b** Ranked inhibition rates of epigenetic compounds in CRC organoids after treatment with each compound at 10 μM for 72 h. FLLL32 is highlighted in red. **c** Chemical structure of FLLL32. **d** Representative images of patient-derived colorectal cancer organoids before and after treatment with 10 μM FLLL32 for 72 h. Scale bar, 50 μm. **e** Inhibition rates of 10 μM FLLL32 in six patient-derived CRC organoid models. **f** IC_50_ values were calculated from cell viability assays of HCT116 and HT29 cells. **g** Cell viability of NCM460 cells treated with the indicated concentrations of FLLL32. Data are presented as the mean ± SD, *n* = 3

We first optimized and validated protocols for cultivating human CRC organoids from tissue samples, establishing a high-throughput 384-well plate screening platform to identify potential anti-CRC compounds (Fig. 1a). Using an epigenetic compound library, we performed organoid-based drug screening to evaluate candidate anti-CRC compounds. The screening library contained 100 compounds with different clinical development stages and target categories. Among these compounds, 56% had not yet entered clinical trials, while the remaining compounds were distributed across phase I-IV clinical stages (Fig. S1a). The major target categories included JAK (18%), Aurora kinase (17%), PARP (16%), and other tumor-associated pathways (Fig. S1b).

The ranked screening profile of the library identified FLLL32 as a highly effective hit in patient-derived CRC organoids (Fig. 1b, Table S1). FLLL32, a curcumin analogue and JAK-STAT pathway inhibitor, was chemically modified with a spiroalkyl ring at the intermediate carbon of curcumin to prevent enolization, thereby improving its solubility and bioavailability (Fig. 1c). We found that FLLL32 effectively inhibited patient-derived CRC organoid growth in all six cases (Fig. 1d, e).

The inhibitory effect of FLLL32 was further evaluated in CRC cell lines. Cell viability assays demonstrated that FLLL32 inhibited HCT116 and HT29 cells in a dose-dependent manner with IC_50_ values of 0.44 μM and 0.33 μM (Fig. 1f). In contrast, FLLL32 showed minimal cytotoxicity toward NCM460 cells at concentrations up to 5 μM (Fig. 1g). Dose-gradient immunoblotting further showed that FLLL32 reduced pSTAT3 levels in a concentration-dependent manner, with marked downregulation of pSTAT3 beginning at 1 μM in HCT116 cells and 1.5 μM in HT29 cells (Fig. S2a, b). Based on these results, 1 μM FLLL32 for HCT116 cells and 1.5 μM FLLL32 for HT29 cells were used in subsequent mechanistic experiments. Given its inhibitory effect on human CRC organoids and CRC cell lines, we further investigated how FLLL32 suppresses CRC growth.

### FLLL32 inhibits proliferation and migration in CRC cells

**Fig. 2:**
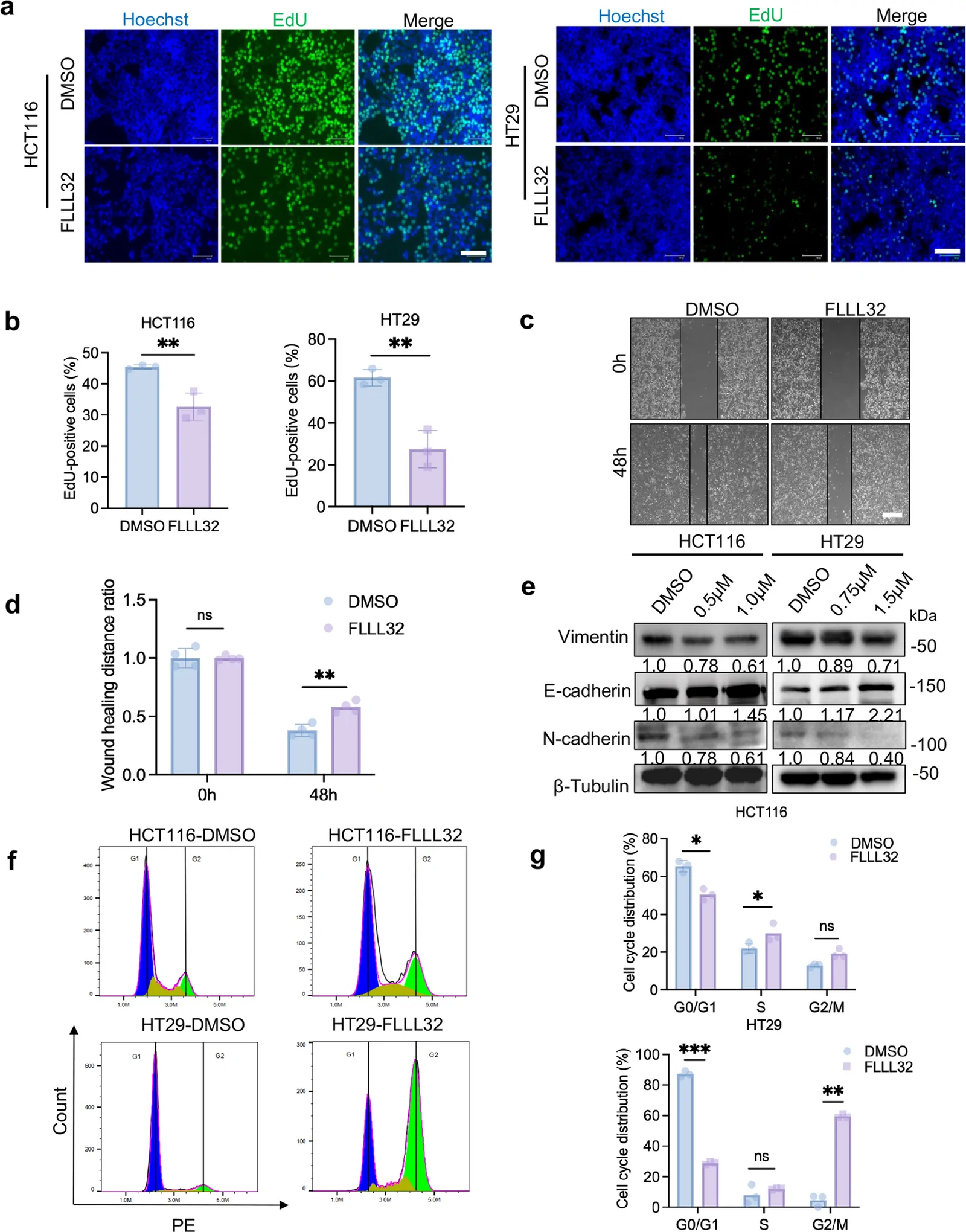
FLLL32 inhibits CRC cell proliferation and migration. **a** Representative EdU staining images of HCT116 and HT29 cells treated with DMSO or FLLL32. Scale bars = 100 μm. **b** Quantification of EdU-positive cells in HCT116 and HT29 cells. **c** Wound-healing images of HCT116 treated with FLLL32 or DMSO. Scale bars = 20 μm. **d** Quantification of wound-healing distance ratio in HCT116 cells. **e** Western blot analysis of vimentin, E-cadherin and N-cadherin in HCT116 and HT29 cells treated with the indicated concentrations of FLLL32. β-Tubulin was used as the loading control. **f** Representative flow cytometry plots showing cell-cycle distribution in HCT116 and HT29 treated with FLLL32 or DMSO for 48 h. **g** Quantification of cell-cycle distribution in HCT116 and HT29 cells. Data are presented as the mean ± SD, *n* = 3. *p < 0.05, **p < 0.01, ***p < 0.001

Based on the inhibitory effect of FLLL32 observed in patient-derived CRC organoids, we next examined whether FLLL32 also suppresses CRC cell proliferation and migration in vitro. EdU incorporation assays showed that FLLL32 significantly reduced the proportion of EdU-positive HCT116 and HT29 cells (Fig. 2a, b). Additionally, wound-healing assays showed that FLLL32 effectively inhibited HCT116 cell migration (Fig. 2c, d). We also analyzed epithelial-mesenchymal transition-associated markers. FLLL32 treatment decreased the expression levels of N-cadherin and vimentin and increased E-cadherin expression in CRC cells (Fig. 2e).

Furthermore, flow cytometric analysis showed that FLLL32 altered cell-cycle distribution in HCT116 and HT29 cells (Fig. 2f, g), suggesting impaired cell-cycle progression after FLLL32 treatment. Consistent with previous studies showing that FLLL32 can induce apoptosis, Annexin V/PI staining showed increased apoptotic populations after FLLL32 treatment in both CRC cell lines (Fig. S3a, b). These results indicate that FLLL32 suppresses CRC cell proliferation and migration, disrupts cell-cycle progression, and promotes apoptotic features.

### FLLL32 induces ferroptosis-associated features in CRC cells

**Fig. 3:**
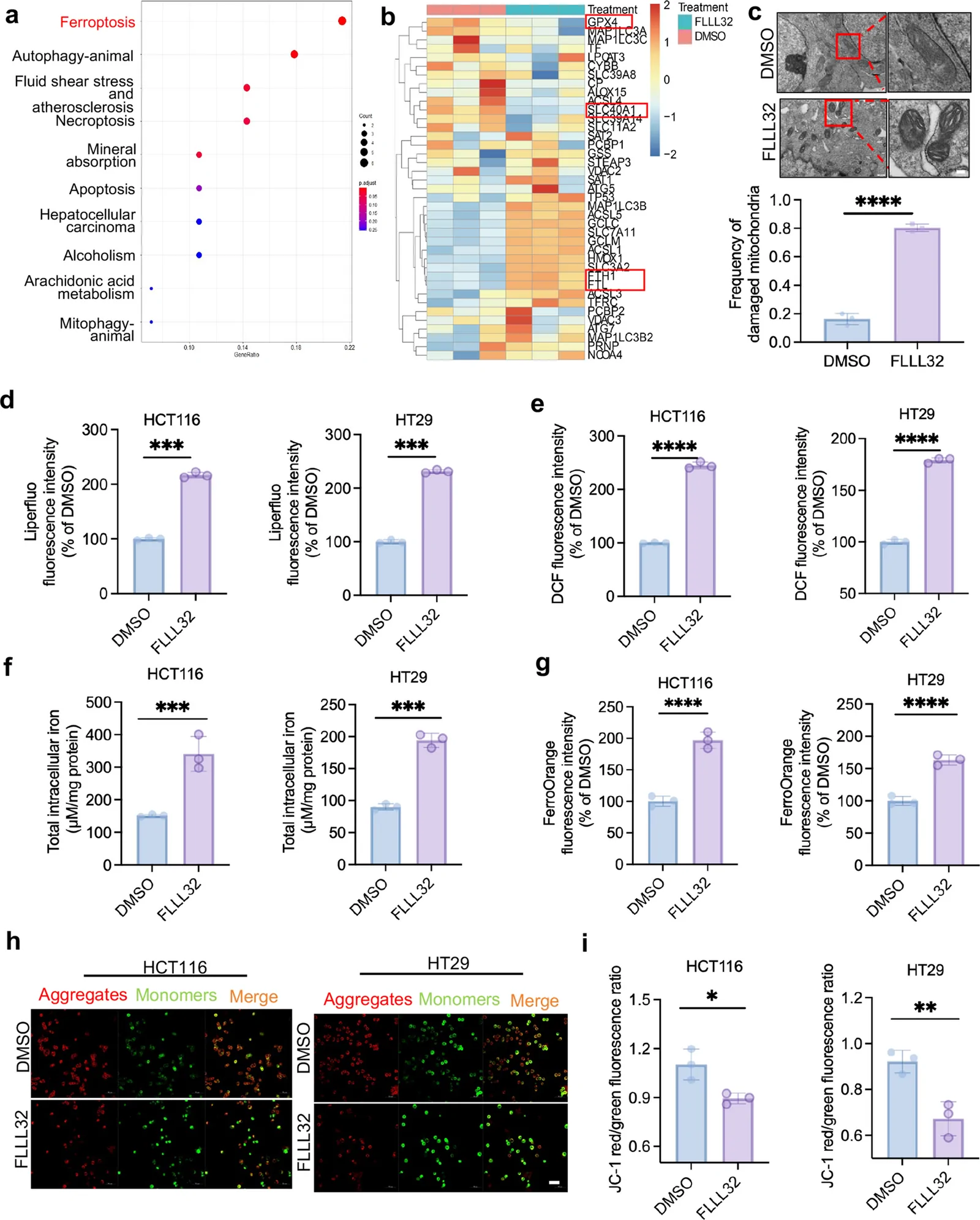
FLLL32 induces ferroptosis-associated lipid peroxidation, iron accumulation, and mitochondrial dysfunction in CRC cells. **a** KEGG pathway enrichment analysis of differentially expressed genes in FLLL32-treated CRC cells. **b** Heatmap showing the expression of ferroptosis-related genes after FLLL32 treatment. **c** Transmission electron microscopy images of HCT116 cells treated with FLLL32 or DMSO, with quantification of damaged mitochondria. Scale bars = 500 nm. **d** Liperfluo fluorescence analysis showing lipid peroxidation levels in HCT116 and HT29 cells treated with FLLL32 or DMSO. **e** DCF fluorescence analysis showing intracellular ROS levels in HCT116 and HT29 cells after FLLL32 treatment. **f** Total intracellular iron content in HCT116 and HT29 cells treated with FLLL32 or DMSO for 48 h. **g** FerroOrange fluorescence analysis showing intracellular Fe^2+^ levels in HCT116 and HT29 cells after FLLL32 treatment. **h** Representative JC-1 staining images showing mitochondrial membrane potential changes in HCT116 and HT29 cells treated with FLLL32. Scale bars = 50 μm. **i** Quantification of JC-1 red/green fluorescence ratio in HCT116 and HT29. Data are presented as the mean ± SD, *n* = 3. *p < 0.05, **p < 0.01, ***p < 0.001, ****p < 0.0001

To further elucidate the mechanisms underlying the anti-CRC activity of FLLL32, we performed RNA sequencing in FLLL32-treated HCT116 cells to identify transcriptional changes and signaling pathways associated with its inhibitory effects. Kyoto Encyclopedia of Genes and Genomes (KEGG) pathway enrichment analysis revealed that, in addition to apoptosis-related changes, the ferroptosis pathway was notably enriched after FLLL32 treatment (Fig. 3a). RNA-seq heatmap analysis further demonstrated that FLLL32 altered the expression of ferroptosis-associated genes, including GPX4, SLC40A1, FTH1, FTL and other genes related to iron homeostasis and oxidative stress (Fig. 3b). Ferroptosis is typically accompanied by characteristic mitochondrial abnormalities [26]. Transmission electron microscopy showed marked mitochondrial changes in HCT116 cells after FLLL32 treatment, including increased membrane density, and diminished or absent cristae (Fig. 3c). These findings suggest that FLLL32 might exert part of its anticancer effects through a ferroptosis-associated mechanism, distinct from its known pro-apoptotic activity.

Several ferroptosis-related biochemical features were then evaluated. Liperfluo staining, which detects lipid peroxidation, showed that FLLL32 increased lipid peroxide accumulation in HCT116 and HT29 cells (Fig. 3d). DCF fluorescence analysis showed increased intracellular ROS levels after FLLL32 treatment (Fig. 3e). Because intracellular iron content plays a critical role in ferroptosis sensitivity, both total intracellular iron and ferrous iron levels were measured. FLLL32 significantly increased total intracellular iron content in CRC cells (Fig. 3f). FerroOrange staining further showed that FLLL32 increased intracellular Fe^2+^ levels (Fig. 3g). JC-1 staining indicated that FLLL32 disrupted the mitochondrial membrane potential, leading to abnormal mitochondrial function and morphology (Fig. 3h, i). Together, these findings indicate that FLLL32 induces ferroptosis-associated lipid peroxidation, iron accumulation, and mitochondrial dysfunction in CRC cells.

### FLLL32 induces a Fer-1-rescuable ferroptotic response in CRC cells

**Fig. 4:**
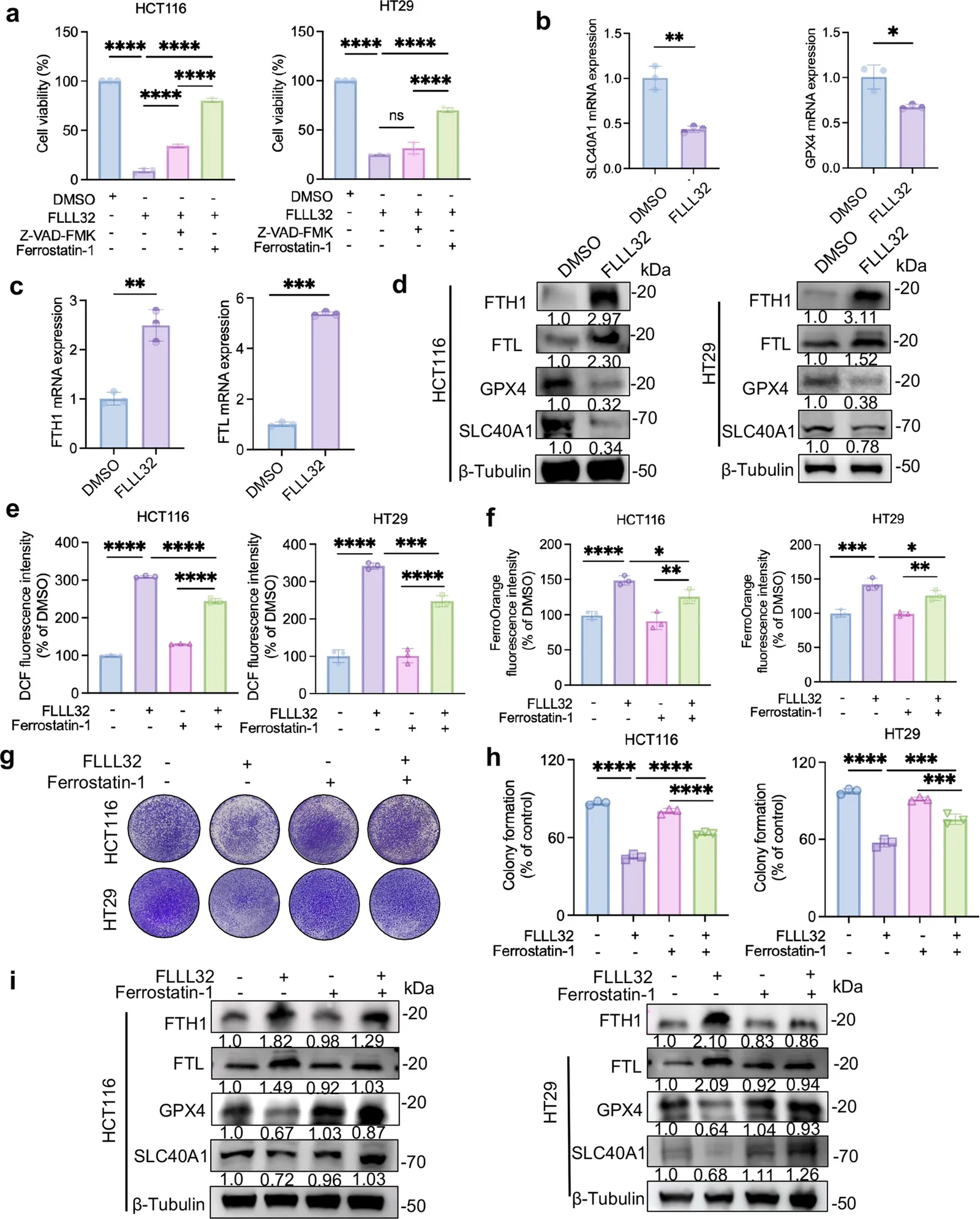
FLLL32 induces a Ferrostatin-1-rescuable ferroptotic response in CRC cells. **a** Cell viability of HCT116 and HT29 cells treated with FLLL32 in the presence or absence of Z-VAD-FMK or Ferrostatin-1. **b**, **c** RT-qPCR analysis of SLC40A1 and GPX4, FTH1 and FTL mRNA expression in HCT116 cells treated with DMSO or FLLL32. **d** Western blot analysis of FTH1, FTL, GPX4 and SLC40A1 in HCT116 and HT29 cells treated with DMSO or FLLL32. β-Tubulin was used as the loading control. **e** DCF fluorescence analysis showing ROS levels in HCT116 and HT29 cells treated with FLLL32 in the presence or absence of Ferrostatin-1. **f** FerroOrange fluorescence analysis showing intracellular Fe^2+^ levels in HCT116 and HT29 cells treated with FLLL32 in the presence or absence of Ferrostatin-1. **g** Colony formation of HCT116 and HT29 cells treated with Ferrostatin-1 and FLLL32. **h** Quantification of colony numbers in HCT116 and HT29 cells. **i** Western blot analysis of FTH1, FTL, GPX4 and SLC40A1 in HCT116 and HT29 cells treated with FLLL32 in the presence or absence of Ferrostatin-1. β-Tubulin was used as the loading control. Data are presented as the mean ± SD, *n* = 3. *p < 0.05, **p < 0.01, ***p < 0.001, ****p < 0.0001

To determine whether ferroptosis functionally contributes to FLLL32-induced cell death, CRC cells were treated with FLLL32 in the absence or presence of Ferrostatin-1 (Fer-1), a ferroptosis inhibitor, or Z-VAD-FMK, an apoptosis inhibitor. FLLL32 markedly reduced cell viability to 9.07% in HCT116 cells and 24.67% in HT29 cells. Z-VAD-FMK only partially restored viability to 34.22% in HCT116 cells and showed minimal protection in HT29 cells, with viability remaining at 31.52%. In contrast, Fer-1 restored cell viability to 80.20% in HCT116 cells and 70.07% in HT29 cells (Fig. 4a). These results indicate that FLLL32-induced cell death is more effectively rescued by ferroptosis inhibition than by apoptosis inhibition.

We examined the expression changes of ferroptosis-related regulators identified from the RNA-seq analysis after FLLL32 treatment. Dose-gradient immunoblotting showed an overall downward trend in GPX4 and SLC40A1 levels with increasing FLLL32 concentrations in CRC cells (Fig. S4a, b). RT-qPCR analysis showed that FLLL32 decreased the mRNA levels of SLC40A1 and GPX4, while increasing FTH1 and FTL expression in HCT116 cells (Fig. 4b, c). Western blot analysis further confirmed that FLLL32 reduced GPX4 and SLC40A1 protein levels and increased FTH1 and FTL protein expression in both HCT116 and HT29 cells (Fig. 4d).

Having shown that Fer-1 markedly rescued FLLL32-induced loss of cell viability, we next examined whether Fer-1 could also attenuate FLLL32-induced ferroptosis-associated cellular responses. Fer-1 attenuated FLLL32-induced DCF fluorescence and FerroOrange fluorescence intensity, indicating that Fer-1 reduced FLLL32-induced ROS accumulation and intracellular Fe^2+^ levels (Fig. 4e, f). Fer-1 also partially restored the colony-forming ability of FLLL32-treated HCT116 and HT29 cells (Fig. 4g, h). Furthermore, Western blot analysis showed that Fer-1 partially attenuated the FLLL32-induced decrease in GPX4 and SLC40A1 expression and partially reduced the ferritin-associated response induced by FLLL32 treatment (Fig. 4i). Collectively, these findings indicate that FLLL32 induced a Fer-1-rescuable ferroptosis phenotype in CRC cells, characterized by reduced cell viability and clonogenicity and increased ROS and Fe^2+^ accumulation. In parallel, FLLL32 treatment was accompanied by changes in the expression of ferroptosis-related proteins, which were partially attenuated by Fer-1.

FTH1 and FTL are ferritin subunits involved in intracellular iron storage and may limit ferroptosis by buffering labile iron. However, FLLL32 increased FTH1 and FTL expression while suppressing GPX4 and SLC40A1, raising the question of why these iron-storage genes were upregulated under ferroptosis-associated stress. We therefore examined the dynamic changes of these ferroptosis-related regulators after FLLL32 treatment. Time-course RT-qPCR and Western blot analyses showed that GPX4 and SLC40A1 were reduced at both the mRNA and protein levels as early as 3 h after FLLL32 treatment and remained suppressed at 6 h and 9 h (Fig. S5a, c). In contrast, ferritin induction occurred later than these changes: FTH1 showed a modest increase beginning at 3 h, whereas FTL upregulation became more evident at 6 h and 9 h (Fig. S5b, c).

To further assess the functional relevance of FTH1 and FTL induction, FTH1 and FTL were knocked down (Fig. S5d). Liperfluo staining showed that FTL or FTH1 knockdown alone produced a modest increase in lipid peroxidation under FLLL32 treatment, whereas combined knockdown of FTH1 and FTL produced the strongest Liperfluo signal (Fig. S5e, f). These results suggest that FTH1 and FTL upregulation is associated with a protective adaptive response during FLLL32-induced ferroptotic stress, and that depletion of FTH1 and FTL further enhances FLLL32-induced lipid peroxidation, thereby promoting ferroptosis.

### FLLL32 targets STAT3 to suppress pSTAT3-mediated GPX4/SLC40A1 transcriptional regulation

STAT3 is a transcription factor that regulates downstream genes involved in tumor survival and therapeutic resistance. Previous studies have reported FLLL32 as a STAT3 inhibitor that suppresses STAT3 Tyr705 phosphorylation and STAT3 DNA-binding activity in multiple cancer cell types, including CRC cells [14]. We examined whether FLLL32 regulates GPX4 and SLC40A1 through STAT3-dependent transcriptional regulation.

**Fig. 5:**
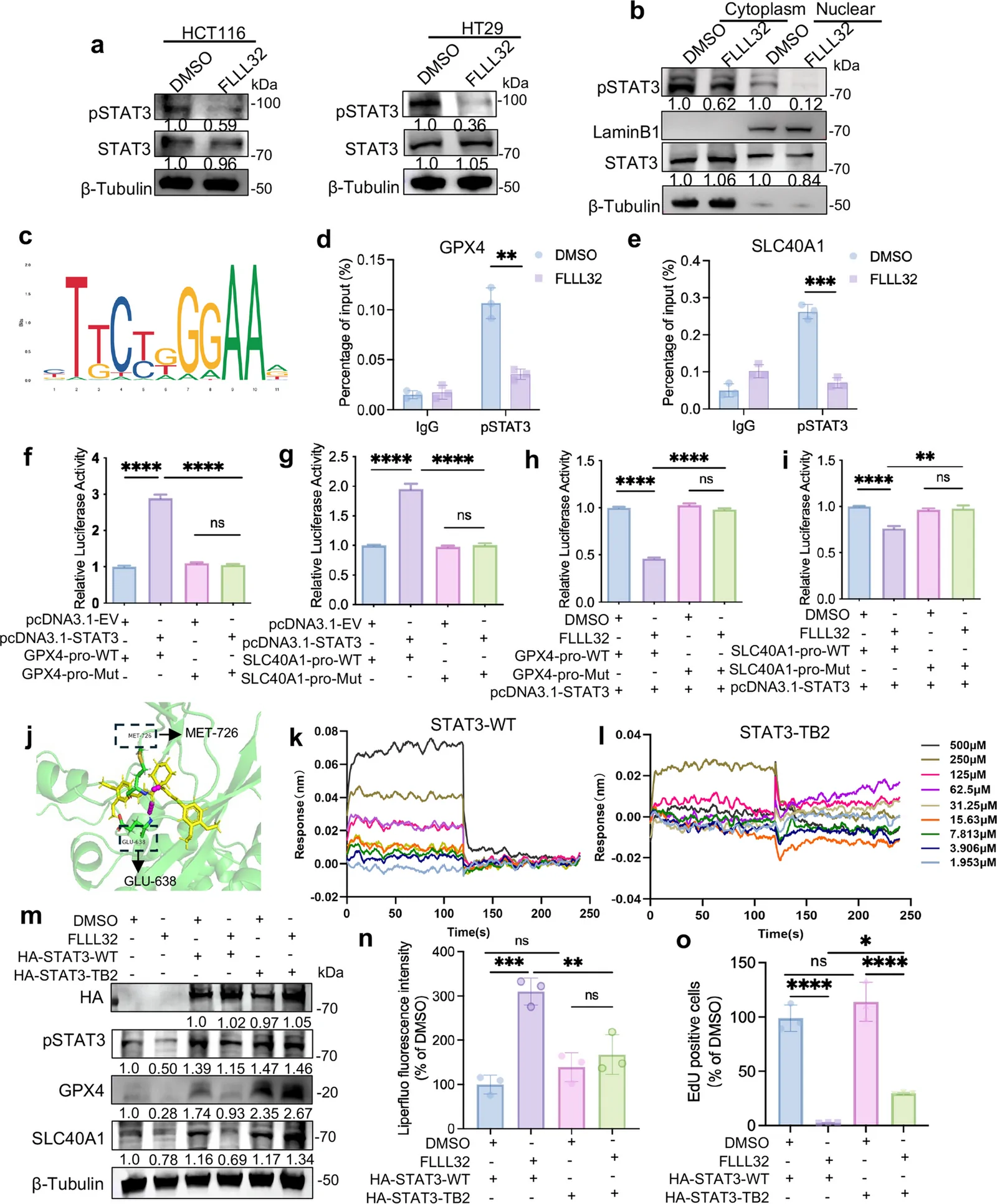
FLLL32 targets STAT3 to suppress pSTAT3-mediated GPX4/SLC40A1 transcriptional regulation. **a** Western blot analysis of pSTAT3, total STAT3, and β-Tubulin in HCT116 and HT29 cells treated with DMSO or FLLL32. β-Tubulin was used as the loading control. **b** Nuclear-cytoplasmic fractionation analysis of pSTAT3 and STAT3 in HCT116 cells treated with DMSO or FLLL32. Lamin B1 and β-Tubulin were used as nuclear and cytoplasmic markers. **c** Predicted STAT3-binding motif within the GPX4 and SLC40A1 promoter regions. **d**, **e** ChIP-qPCR analysis of pSTAT3 occupancy at GPX4 and SLC40A1 promoter regions in HCT116 cells treated with DMSO or FLLL32. IgG was used as the negative control. **f**, **g** Promoter luciferase assays showing the activity of wild-type or mutant GPX4 and SLC40A1 promoters in cells transfected with control or STAT3 expression plasmids. **h**, **i** Dual-luciferase reporter assays of wild-type or STAT3-binding motif-mutant GPX4 (h) and SLC40A1 (i) promoter reporters in STAT3-overexpressing cells after DMSO or FLLL32 treatment. Mut indicates mutation of the predicted STAT3-binding motif. **j** Molecular docking model showing the predicted interaction between FLLL32 and STAT3 at Glu638 and Met726. **k**, **l** Bio-layer interferometry analysis of the binding between FLLL32 and purified STAT3-WT and STAT3-TB2 proteins. **m** Western blot analysis of HA, pSTAT3, SLC40A1, GPX4 in STAT3-WT- or STAT3-TB2-expressing cells treated with DMSO or FLLL32. β-Tubulin was used as the loading control. **n** Quantification of Liperfluo fluorescence intensity showing lipid peroxidation in HA-STAT3-WT- or HA-STAT3-TB2-expressing cells after DMSO or FLLL32 treatment. **o** Quantification of relative EdU-positive cells in HCT116 cells expressing HA-STAT3-WT or HA-STAT3-TB2 after DMSO or FLLL32 treatment. Data are presented as the mean ± SD, *n* = 3. *p < 0.05, **p < 0.01, ***p < 0.001, ****p < 0.0001

Western blot analysis showed that FLLL32 markedly reduced STAT3 (Tyr705) phosphorylation in HCT116 and HT29 cells, while total STAT3 levels remained unchanged (Fig. 5a). Nuclear-cytoplasmic fractionation showed that FLLL32 reduced pSTAT3 levels in both the cytoplasmic and nuclear fractions, with the reduction appearing more evident in the nuclear fraction. Lamin B1 and β-Tubulin were used as nuclear and cytoplasmic markers (Fig. 5b). These data indicate that FLLL32 suppresses STAT3 phosphorylation and attenuates nuclear pSTAT3 signaling in CRC cells.

We then examined whether FLLL32 affected pSTAT3 occupancy at the GPX4 and SLC40A1 promoters. Sequence analysis identified putative STAT3-binding motifs within the GPX4 and SLC40A1 promoter regions (Fig. 5c). Chromatin immunoprecipitation quantitative PCR (ChIP-qPCR) analysis showed that pSTAT3 was enriched at the promoter regions of GPX4 and SLC40A1, whereas FLLL32 treatment significantly reduced pSTAT3 occupancy at both promoter regions (Fig. 5d, e). Promoter luciferase assays further showed that STAT3 increased the activities of wild-type SLC40A1 and GPX4 promoters, whereas mutation of the predicted STAT3-binding motifs abolished this transcriptional activation (Fig. 5f, g). Importantly, FLLL32 significantly suppressed the activity of wild-type GPX4 and SLC40A1 promoter reporters, while this inhibitory effect was largely abolished in the corresponding STAT3-binding motif-mutant reporters (Fig. 5h, i). These results suggest that STAT3 promotes GPX4 and SLC40A1 transcriptional activation through the predicted STAT3-binding motifs, and that FLLL32 suppresses this pSTAT3-mediated GPX4/SLC40A1 transcriptional regulation.

To further investigate the interaction between STAT3 and FLLL32, we performed a computer-aided molecular docking analysis. The docking model predicted that FLLL32 interacts with STAT3 near Glu638 and Met726 (Fig. 5j). We then purified wild-type (WT) STAT3 and STAT3-TB2 mutant with alanine substitutions at Glu638 and Met726 (within 3 Å of the predicted binding site). Bio-layer interferometry analysis showed concentration-dependent binding between FLLL32 and purified STAT3-WT protein (Fig. 5k), whereas the STAT3-TB2 mutant showed a markedly weaker binding response (Fig. 5l). These results indicate that FLLL32 directly binds to STAT3 and suggest that Glu638 and Met726 are important residues involved in the FLLL32-STAT3 interaction.

Western blot results showed that FLLL32 decreased pSTAT3, GPX4, and SLC40A1 levels in control and HA-STAT3-WT-expressing cells, whereas HA-STAT3-TB2 expression partially preserved pSTAT3, GPX4, and SLC40A1 levels after FLLL32 treatment (Fig. 5m). Consistently, compared with HA-STAT3-WT, HA-STAT3-TB2 attenuated FLLL32-induced lipid peroxidation, as indicated by decreased Liperfluo fluorescence intensity (Fig. 5n). In addition, HA-STAT3-TB2 partially rescued the growth-inhibitory effect of FLLL32, as reflected by increased EdU-positive cells compared with HA-STAT3-WT under FLLL32 treatment (Fig. 5o). Without FLLL32 treatment, HA-STAT3-WT and HA-STAT3-TB2 showed no significant differences in Liperfluo fluorescence intensity or the proportion of EdU-positive cells. Collectively, these results identified Glu638 and Met726 as important residues involved in the FLLL32-STAT3 interaction and demonstrated that FLLL32 targets STAT3 to suppress pSTAT3-mediated GPX4/SLC40A1 transcriptional regulation, thereby contributing to the FLLL32-induced ferroptotic process in CRC cells.

### Restoration of the STAT3-GPX4/SLC40A1 axis attenuates FLLL32-induced ferroptosis in CRC cells

**Fig. 6:**
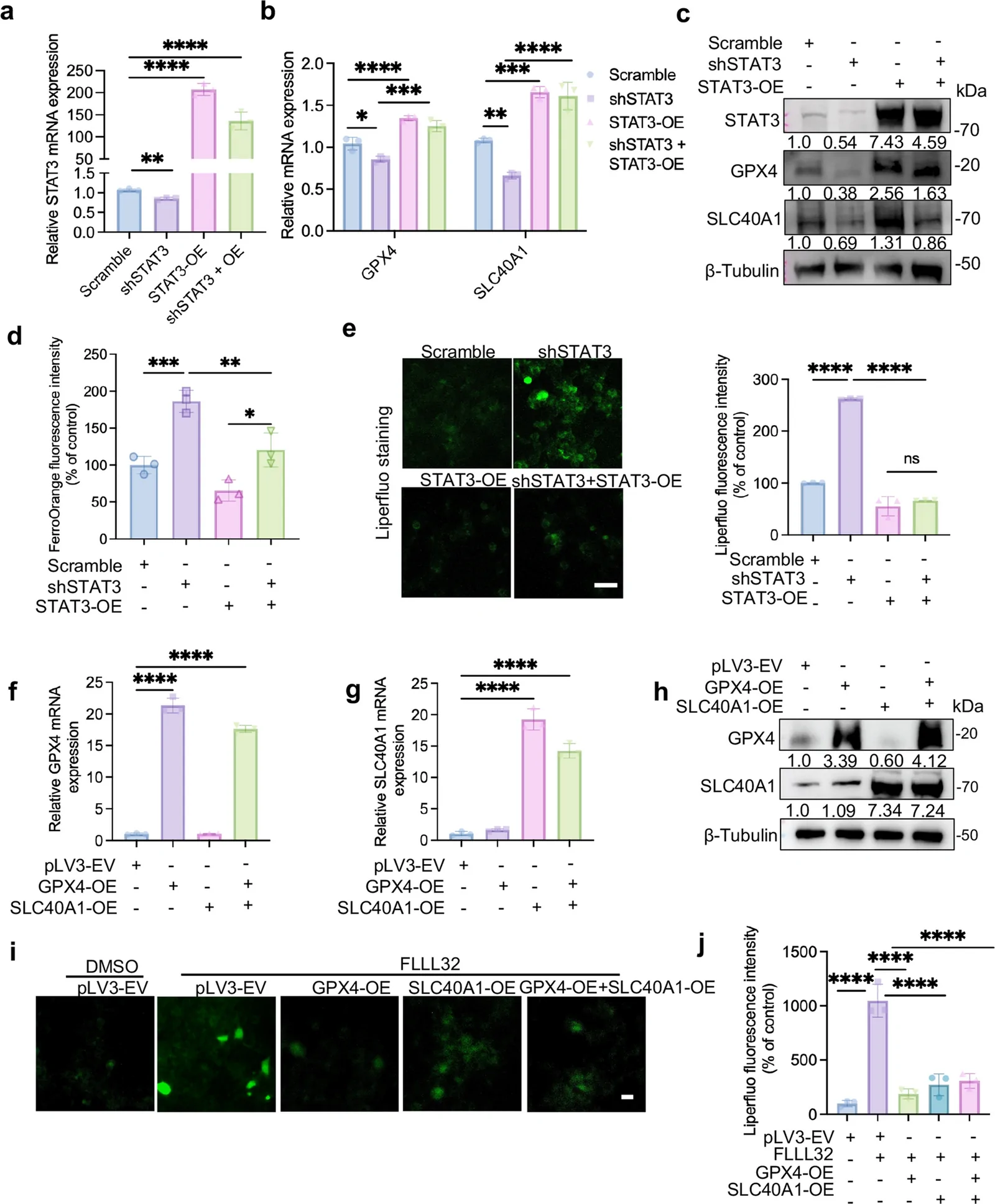
Restoration of the STAT3-GPX4/SLC40A1 axis attenuates FLLL32-induced ferroptosis in CRC cells. **a**, **b** RT-qPCR analysis of STAT3, GPX4 and SLC40A1 mRNA expression in scramble, shSTAT3, STAT3-overexpressing, and shSTAT3 cells with STAT3 re-expression. **c** Western blot analysis of STAT3, GPX4, and SLC40A1 in scramble, shSTAT3, STAT3-overexpressing, and shSTAT3 cells with STAT3 re-expression. β-Tubulin was used as the loading control. **d** FerroOrange staining quantification of intracellular Fe^2+^ levels in scramble, shSTAT3, STAT3-overexpressing, and shSTAT3 cells with STAT3 re-expression. **e** Representative Liperfluo staining images and quantification of Liperfluo fluorescence intensity in HCT116 cells with STAT3 knockdown or STAT3 re-expression. Scale bar, 50 μm. **f**, **g** RT-qPCR analysis confirming GPX4 and/or SLC40A1 overexpression in HCT116 cells. **h** Western blot analysis confirming GPX4 and/or SLC40A1 overexpression in HCT116 cells. β-Tubulin was used as the loading control. **i** Representative Liperfluo staining images of HCT116 cells overexpressing GPX4, SLC40A1, or both after FLLL32 treatment. Scale bar, 10 μm. **j** Quantification of Liperfluo fluorescence intensity in HCT116 cells overexpressing GPX4, SLC40A1, or both after FLLL32 treatment. Data are presented as the mean ± SD, *n* = 3. *p < 0.05, **p < 0.01, ***p < 0.001, ****p < 0.0001

Since STAT3 was identified as a functional target of FLLL32, we next examined whether restoration of the STAT3-GPX4/SLC40A1 axis could attenuate ferroptosis-associated changes in CRC cells. STAT3 knockdown reduced GPX4 and SLC40A1 expression at both the mRNA and protein levels, whereas STAT3 overexpression increased GPX4 and SLC40A1 expression. Re-expression of STAT3 in STAT3-depleted cells partially restored GPX4 and SLC40A1 expression (Fig. 6a-c). Consistent with these changes, STAT3 knockdown increased intracellular Fe^2+^ accumulation and lipid peroxidation, while STAT3 re-expression attenuated these increases induced by STAT3 depletion (Fig. 6d, e).

In contrast, the ferritin-related genes FTL and FTH1 were modestly increased after STAT3 knockdown, and this induction was not reversed by STAT3 re-expression (Fig. S5g, h). These findings suggest that FTH1 and FTL upregulation may represent a compensatory ferritin response associated with STAT3 depletion-induced ferroptotic stress, rather than a direct downstream rescue effect of STAT3 restoration. Together, these results indicate that STAT3 positively regulates GPX4/SLC40A1 expression and limits ferroptotic stress in CRC cells.

To further determine whether STAT3 restoration could counteract FLLL32-induced ferroptosis-associated changes, STAT3 was overexpressed in FLLL32-treated CRC cells. STAT3 overexpression partially reversed the FLLL32-induced reduction of GPX4 and SLC40A1 and decreased FLLL32-induced DCF-positive signals in HCT116 and HT29 cells (Fig. S6a-c). These findings further support that STAT3 restoration attenuates FLLL32-induced oxidative stress and downstream ferroptosis-associated changes.

We then examined whether restoration of the downstream ferroptosis-defense regulators GPX4 and SLC40A1 could attenuate FLLL32-induced ferroptotic stress. RT-qPCR and Western blot analyses confirmed the overexpression of GPX4 and SLC40A1 (Fig. 6f-h). Liperfluo staining showed that overexpression of GPX4, SLC40A1, or both markedly reduced FLLL32-induced lipid peroxidation compared with FLLL32-treated pLV3-EV control cells (Fig. 6i, j). These data suggest that restoration of GPX4 and SLC40A1 attenuates FLLL32-induced ferroptotic stress.

Collectively, these results support the functional involvement of the STAT3-GPX4/SLC40A1 axis in FLLL32-induced ferroptosis in CRC cells.

### FLLL32 suppresses CRC xenograft growth in vivo

**Fig. 7:**
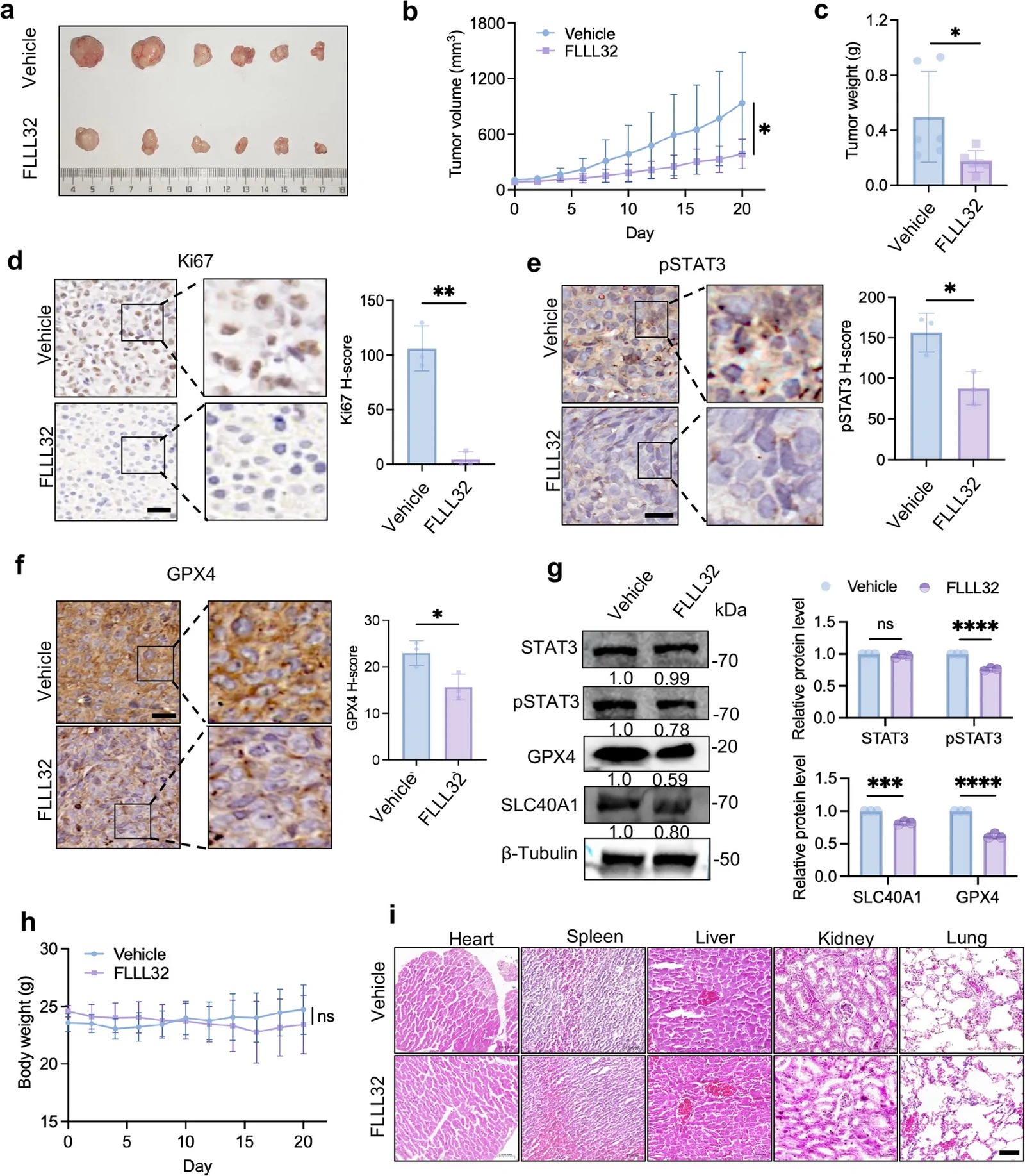
FLLL32 suppresses CRC xenograft growth and inhibits the STAT3-GPX4/SLC40A1 axis in vivo*.* **a** Images of xenograft tumors from vehicle- and FLLL32-treated mice (*n* = 6). **b** Tumor growth curves of HCT116 xenografts treated with vehicle or FLLL32 (*n* = 6). **c** Statistics of tumor weight (*n* = 6). **d**-**f** Ki67, pSTAT3 and GPX4 immunohistochemical staining images and H-score quantification in xenograft tumors (*n* = 3). Scale bars = 25 μm. **g** Western blot analysis and quantification of STAT3, pSTAT3, GPX4, and SLC40A1 in xenograft tumor tissues. β-Tubulin was used as the loading control (*n* = 3). **h** Body weight changes during vehicle or FLLL32 treatment. **i** Representative H&E staining images of major organs, including heart, spleen, liver, kidney, lung from vehicle- and FLLL32-treated mice. Scale bars = 50 μm. Data are presented as the mean ± SD. *p < 0.05, **p < 0.01, ***p < 0.001, ****p < 0.0001

A subcutaneous xenograft model was established in BALB/c-nu/nu mice using HCT116 cells. Mice received daily intraperitoneal injections of either vehicle or FLLL32 (50 mg/kg/day). FLLL32 significantly inhibited tumor growth, as shown by reduced tumor volume and tumor weight compared with the vehicle group (Fig. 7a-c).

Immunohistochemical analysis of tumor tissues confirmed that FLLL32 treatment markedly suppressed tumor cell proliferation, as indicated by decreased Ki67 staining and reduced Ki67 H-score (Fig. 7d). Furthermore, FLLL32 treatment ​also reduced​ the pSTAT3 and GPX4 staining in tumor tissues, and H-score quantification confirmed significant decreases in these markers (Fig. 7e, f). Western blot analysis corroborated these findings, showing that FLLL32 treatment ​significantly decreased​ pSTAT3, GPX4 and SLC40A1 levels, whereas total STAT3 was not significantly changed (Fig. 7g). Body weight monitoring showed no significant difference between the vehicle and FLLL32-treated groups throughout the treatment period (Fig. 7h). Histopathological examination of major organs, including the heart, spleen, liver, kidney, and lung, did not reveal obvious histological toxicity in major organs (Fig. 7i). These results indicate that FLLL32 suppresses CRC xenograft growth and inhibits the STAT3-GPX4/SLC40A1 axis in vivo without causing overt body-weight loss or obvious histological injury in major organs under the tested conditions.

## Discussion

In this study, we identified FLLL32 as an active anti-CRC compound using a patient-derived CRC organoid screening platform and defined a ferroptosis-associated mechanism underlying its anti-tumor activity. FLLL32 suppressed CRC organoid growth, CRC cell proliferation and migration, and xenograft tumor growth. Meanwhile, FLLL32 induced mitochondrial injury, iron accumulation, lipid peroxidation, and Fer-1-rescuable ferroptotic cell death. These findings suggest that FLLL32 exerts anti-CRC activity not only by suppressing tumor growth but also by inducing ferroptosis-associated cellular damage.

FLLL32 has generally been considered a STAT3-targeting compound with pro-apoptotic activity [27]. Consistent with previous studies, we also observed apoptotic features after FLLL32 treatment, indicating that apoptosis contributes to the cellular response induced by FLLL32. However, our inhibitor-rescue experiments showed that apoptosis was not the only regulated cell death mode involved in FLLL32-induced cell death. Compared with the apoptosis inhibitor Z-VAD-FMK, the ferroptosis inhibitor Fer-1 more effectively restored cell viability. Fer-1 also partially restored colony-forming ability and reduced FLLL32-induced ROS and Fe^2+^ accumulation. Thus, unlike previous studies that mainly emphasized STAT3 inhibition and apoptosis, our study expands the functional understanding of FLLL32 by identifying ferroptosis as an important component of its anti-CRC activity. Recent CRC-related studies have shown that ferroptosis can be induced by compounds that disrupt antioxidant defense or alter iron homeostasis [28–31]. In line with these studies, FLLL32 induced iron accumulation and lipid peroxidation; however, our findings further link this ferroptotic response to direct STAT3 targeting and suppression of pSTAT3-mediated GPX4/SLC40A1 transcriptional regulation. This extends recent evidence that STAT3/GPX4-associated signaling can regulate ferroptosis in CRC. Therefore, FLLL32-induced CRC cell death is better understood as a multilayered regulated cell death response in which apoptosis and ferroptosis coexist, with ferroptosis constituting an important functional component.

Mechanistically, our data support a model in which FLLL32 induces ferroptosis by directly targeting STAT3 and suppressing pSTAT3-mediated GPX4/SLC40A1 transcriptional regulation. FLLL32 reduced STAT3 Tyr705 phosphorylation, nuclear pSTAT3 levels, pSTAT3 occupancy at the GPX4 and SLC40A1 promoters, and STAT3-induced GPX4/SLC40A1 promoter reporter activity. Molecular docking and BLI analyses further supported a direct interaction between FLLL32 and STAT3, whereas mutation of Glu638 and Met726 weakened the FLLL32-STAT3 interaction and attenuated FLLL32-induced GPX4/SLC40A1 suppression, lipid peroxidation, and the growth-inhibitory effect of FLLL32. Functionally, STAT3 re-expression attenuated STAT3 depletion-induced Fe^2+^ accumulation and lipid peroxidation and partially counteracted FLLL32-induced GPX4/SLC40A1 suppression and oxidative stress. Moreover, GPX4/SLC40A1 overexpression reduced FLLL32-induced lipid peroxidation and attenuated ferroptotic stress. These findings collectively support the functional involvement of the STAT3-GPX4/SLC40A1 axis in FLLL32-induced ferroptosis in CRC cells.

A key feature of this mechanism is the disruption of iron-lipid peroxide homeostasis. The suppression of GPX4 and SLC40A1 should not be interpreted as two isolated events; rather, these changes may jointly weaken the cellular defense against iron-dependent lipid peroxidation. GPX4 downregulation impairs lipid peroxide detoxification, whereas SLC40A1 downregulation may limit iron export and increase the intracellular labile iron pool. Increased labile iron can further promote ROS generation and lipid oxidation, thereby amplifying ferroptotic damage when lipid peroxide clearance is impaired [32]. In addition, FTH1 and FTL upregulation occurred after the early suppression of GPX4/SLC40A1, and FTH1/FTL knockdown further enhanced Liperfluo signals. These results suggest that ferritin induction is more likely an adaptive iron-buffering response during FLLL32-induced ferroptotic stress rather than a contradiction to ferroptosis induction.

Our findings may also have implications for ferroptosis-oriented CRC research. First, the patient-derived CRC organoid screening platform provided a clinically relevant system for identifying FLLL32 as an active anti-CRC candidate [33, 34]. Second, our findings connect STAT3 inhibition with ferroptosis regulation through GPX4 and SLC40A1, providing a mechanistic explanation for how FLLL32 disrupts iron-lipid peroxide homeostasis. Third, because ferroptosis has been implicated in therapeutic response and drug tolerance in CRC [35, 36], the STAT3-GPX4/SLC40A1 axis may represent a potential vulnerability for ferroptosis-oriented therapeutic strategies. Thus, FLLL32 may have value not only as a STAT3-targeting compound but also as a candidate agent for inducing ferroptosis-associated tumor suppression.

Despite these findings, several questions remain to be addressed. GPX4 and SLC40A1 expression was not examined in clinical CRC samples, which limits the assessment of their clinical relevance to FLLL32 sensitivity. In addition, although FLLL32 suppressed tumor growth in the xenograft model, this model was established in immunodeficient mice and therefore could not evaluate the interaction between FLLL32-induced ferroptosis and anti-tumor immunity. Given the reported crosstalk between ferroptosis and immune responses, future studies should investigate whether FLLL32-induced ferroptosis influences immune-related responses [37, 38]. Whether FLLL32-induced ferroptosis can overcome treatment tolerance or acquired drug resistance remains to be determined, although recent studies have suggested that ferroptosis activation may overcome acquired drug resistance or sensitize drug-tolerant CRC models to chemotherapy. Moreover, the toxicity evaluation in this study was mainly based on histopathological examination of major organs. More comprehensive safety assessments, including hematological analysis, serum biochemical tests for liver and kidney function, and longer-term toxicity observation, are needed to further evaluate the systemic toxicity profile of FLLL32. Validation in normal colon organoids is needed to further assess the potential toxicity of FLLL32. Finally, although BLI and mutational analyses support a direct interaction between FLLL32 and STAT3, this study lacks cellular target engagement assays, such as CETSA or related thermal shift analyses, to confirm FLLL32-STAT3 binding in a cellular context. Additional structural and cellular target engagement studies are therefore needed to further clarify the precise binding mode and intracellular target engagement of FLLL32 with STAT3.

**Fig. 8:**
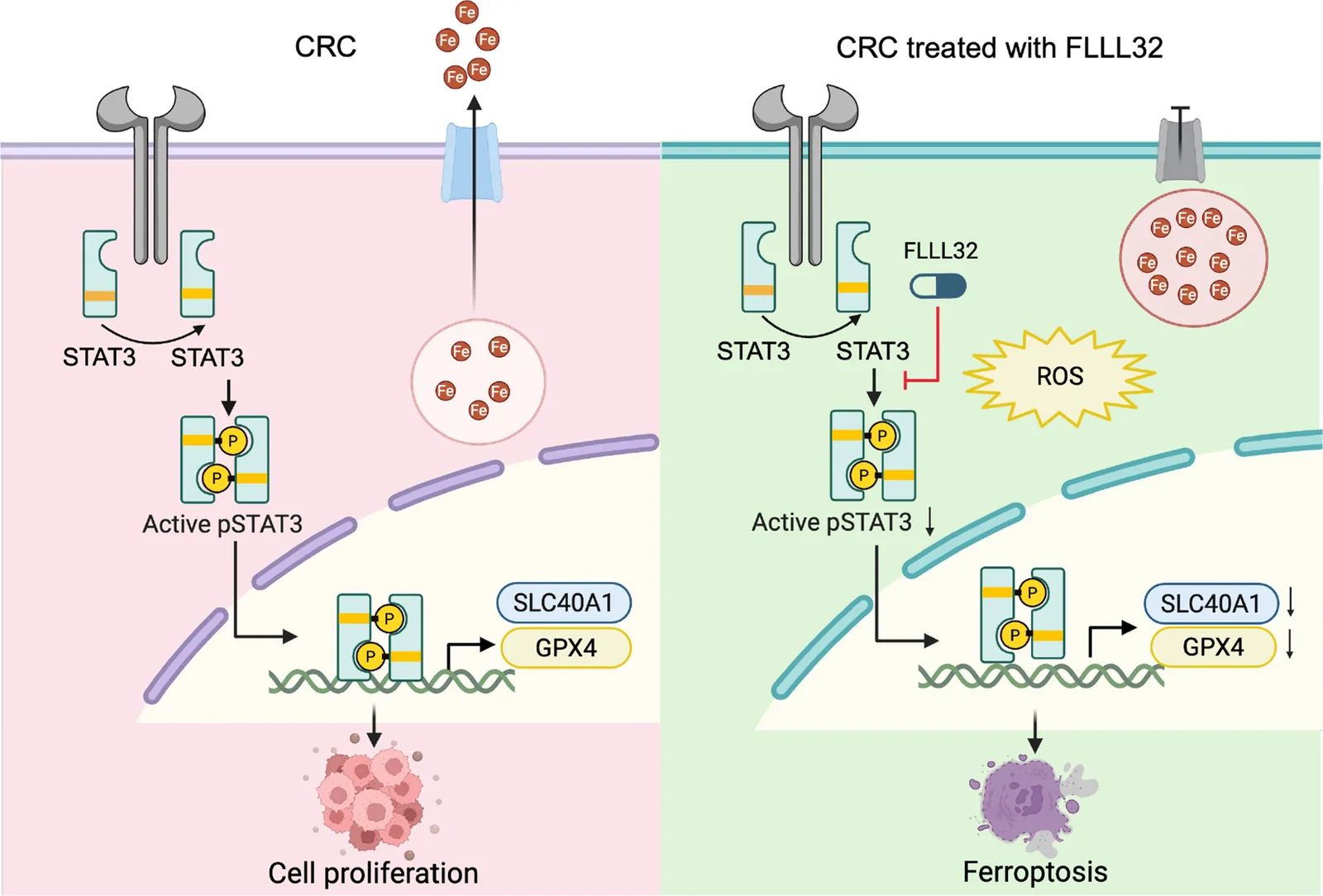
Working model of FLLL32-induced ferroptosis in CRC cells. In untreated CRC cells, STAT3 undergoes Tyr705 phosphorylation and subsequent nuclear signaling to support GPX4 and SLC40A1 transcription, thereby maintaining iron-lipid peroxide homeostasis and promoting CRC cell survival. Upon FLLL32 treatment, FLLL32 targets STAT3 and suppresses its phosphorylation and nuclear signaling, leading to reduced GPX4 and SLC40A1 expression. This impairs lipid peroxide detoxification and iron homeostasis, resulting in ROS accumulation, intracellular Fe^2+^ accumulation, and lipid peroxidation, ultimately promoting ferroptosis in CRC cells

Together, this study identifies FLLL32 as an active anti-CRC compound and defines a STAT3-GPX4/SLC40A1-dependent mechanism for FLLL32-induced ferroptosis. FLLL32 directly targets STAT3, suppresses STAT3 Tyr705 phosphorylation and pSTAT3-dependent nuclear signaling, reduces GPX4/SLC40A1 transcriptional regulation, and disrupts iron-lipid peroxide homeostasis, thereby promoting iron-dependent lipid peroxidation and ferroptotic cell death (Fig. 8). These findings provide a mechanistic basis for the anti-CRC activity of FLLL32 and highlight the STAT3-GPX4/SLC40A1 axis as a relevant ferroptosis-defense pathway that warrants further investigation in CRC.

## Materials and methods

### Cell lines

HCT116, HT29, NCM460, and HEK293T cells were purchased from Shanghai Cell Bank. HCT116, HT29, and HEK293T cells were cultured in Dulbecco’s modified Eagle’s medium (DMEM) (HyClone, AG29629575) supplemented with 10% fetal bovine serum (FBS) (ExCell Bio, FSP500) and 100 U/ml penicillin/streptomycin (Beyotime, C0222) at 37 ℃ in a humidified incubator (Thermo Fisher Scientific) with 5% CO_2_. NCM460 cells were cultured in RPMI 1640 medium (Gibco, C11875500) supplemented with 10% FBS and 100 U/ml penicillin/streptomycin. All cell lines were confirmed by short tandem repeat (STR) analysis before use.

### Patient-derived colorectal cancer organoid culture and drug screening

Fresh colorectal cancer surgical specimens were obtained from 6 patients with pathologically confirmed colorectal adenocarcinoma who underwent surgical resection at West China Hospital of Sichuan University. All patients had not received preoperative radiotherapy or chemotherapy. The collected specimens were washed with PBS containing penicillin–streptomycin, minced into small fragments, and enzymatically digested with collagenase I and collagenase IV at 37 °C. After digestion and centrifugation, the cell pellet was further dissociated with trypsin. The dissociated cells were collected, resuspended in Matrigel with CRC organoid medium (BioGenous, K2103-CR).

For organoid-based drug screening, established CRC organoids were seeded into 384-well plates at 2,000 cells per well in Matrigel. Compounds from the selected compound library described above were added to the culture medium. DMSO-treated organoids served as vehicle controls. Bright-field images were acquired before treatment and after 72 h of compound exposure. Organoid responses were evaluated based on morphological disruption after treatment. Organoids with collapse of the three-dimensional structure, marked fragmentation, or conversion into cell debris were defined as fragmented organoids. The organoid inhibition rate was calculated as the percentage of fragmented/disrupted organoids among the total number of organoids in each well: inhibition rate (%) was calculated as follows: fragmented organoids/total organoids ×100%.

### Reagents and drug treatment

FLLL32 was purchased from Selleck (S7259) and dissolved in DMSO. Ferrostatin-1 (MedChemExpress, HY-100579) and Z-VAD-FMK (MedChemExpress, HY-16658B) were dissolved in DMSO. The compounds used for organoid-based drug screening were from the Epigenetics Compound Library (Selleckchem, L1900). For the initial organoid-based drug screening, all compounds were tested at a final concentration of 10 μM for 72 h. For patient-derived colorectal cancer organoid experiments, organoids were treated with 10 μM FLLL32 for 72 h. For cell-line experiments, HCT116 and HT29 cells were treated with 1 μM and 1.5 μM FLLL32, respectively, for 48 h, unless otherwise specified. In inhibitor-rescue experiments, Fer-1 and Z-VAD-FMK were used at final concentrations of 2 μM and 20 μM. DMSO-treated cells or organoids served as vehicle controls.

### Flow cytometry assays

Cell cycle distribution was determined by Cell Cycle Analysis Kit (Beyotime, C1052). Apoptosis was determined with an Annexin V-FITC Apoptosis Detection Kit (Beyotime, C1062). The mitochondrial membrane potential (MMP) was determined with a JC-1 assay kit (Beyotime, C2006). Reactive oxygen species (ROS) were analyzed using a ROS assay kit (Beyotime, S0033S). These experiments were performed based on the corresponding manufacturer’s instructions. Stained cells (> 10,000) were examined using a FACS flow cytometer (Beckman CytoFLEX, Beckman Coulter Life Sciences, USA). Data were analyzed using FlowJo software.

### Fluorescence imaging assays

Intracellular ferrous ions were detected using a FerroOrange assay kit (DOJINDO, F374), and cellular lipid peroxidation was assessed using a Liperfluo assay kit (DOJINDO, L248). These assays were performed according to the manufacturers’ instructions. Fluorescence images were acquired under identical exposure settings using an Olympus IX73 fluorescence microscope (Olympus, Japan). The fluorescence intensity of FerroOrange and Liperfluo was quantified using ImageJ software in randomly selected fields and normalized to the control group.

### Total intracellular iron measurement

Total intracellular iron content was measured using a Cell Total Iron Content Colorimetric Assay Kit (Applygen Technologies Inc, E1042) according to the manufacturer’s instructions. Briefly, cells were lysed, and the supernatant was collected as the sample. Samples and standards were incubated with the reaction buffer and potassium permanganate solution at 60 °C for 1 h. After cooling to room temperature, iron ion detection reagent was added and incubated for 30 min. Absorbance was measured at 540–580 nm using a microplate reader. Total iron levels were normalized to total protein concentration determined by BCA assay.

### RNA sequencing

Total RNA was extracted from DMSO- and FLLL32-treated CRC cells using a Total RNA Kit (FORGENE, RE03111). RNA quality was assessed before library construction. Sequencing libraries were generated and sequenced on an Illumina HiSeq 2000 platform. Raw reads were trimmed to remove adaptor sequences and low-quality reads. Clean reads were aligned to the human reference genome hg38 using STAR. Gene-level read counts were generated using featureCounts. Differentially expressed genes were identified using DESeq2. Genes with |log2(fold change)|> 1 and adjusted *P* value < 0.05 were considered differentially expressed. KEGG pathway enrichment analysis was performed to identify biological pathways altered after FLLL32 treatment.

### Western blot

Proteins from cells or tumor tissues were extracted using RIPA buffer containing protease and phosphatase inhibitors. Protein concentrations were determined using a BCA assay. Equal amounts of protein were separated by SDS-PAGE, transferred to PVDF membranes, and incubated with primary antibodies followed by HRP-conjugated secondary antibodies. Signals were detected using ECL substrate and quantified by ImageJ. For quantification, pSTAT3 was normalized to total STAT3, while other proteins were normalized to β-Tubulin. Nuclear and cytoplasmic proteins were normalized to Lamin B1 and β-Tubulin, respectively. All values were normalized to the corresponding DMSO control.

Primary antibodies used for immunoblot analysis were as follows: β-Tubulin (ZSGB-BIO, TA347064), STAT3 (HUABIO, ET1603-40), Phospho-STAT3 (Tyr705) (CST, 9145S), GPX4 (HUABIO, ET1706-45), SLC40A1 (CST, 80672T), FTH1 (Abclonal, A1144), FTL (Abclonal, A1768), Lamin B1 (Abclonal, A11495), E-cadherin (Proteintech, 20874-1-AP), N-cadherin (Proteintech, 22018-1-AP), Vimentin (Proteintech, 10366-1-AP), and HA-tag (Proteintech, 66006-2-Ig). HRP-conjugated goat anti-rabbit IgG (ZSGB-BIO, ZF-0511) and HRP-conjugated goat anti-mouse IgG (ZSGB-BIO, ZB-5305) were used as secondary antibodies.

### ChIP-qPCR assay

ChIP assay was performed according to the protocol published previously [39]. Cells were cross-linked with 1% formaldehyde and quenched with glycine. Chromatin was extracted and sonicated to approximately 500 bp fragments. The chromatin lysates were immunoprecipitated with anti-Phospho-STAT3 (Tyr705) antibody (CST, 9145) or control IgG at 4 °C overnight, followed by incubation with Protein G agarose beads. After washing and reversal of cross-linking, immunoprecipitated DNA was purified and analyzed by qPCR using primers targeting the predicted STAT3-binding regions in the GPX4 and SLC40A1 promoters. Data were presented as the percentage of input. Primer sequences are listed in Table S2.

### Promoter luciferase reporter assay

Wild-type and mutant promoter fragments of SLC40A1 and GPX4 containing the predicted STAT3-binding sites were synthesized and cloned into the pGL3-basic luciferase reporter vector. The STAT3 coding sequence was cloned into the pcDNA3.1 expression vector. HEK293T cells were co-transfected with the indicated pGL3 reporter plasmids, pcDNA3.1-STAT3 or empty vector, and the pRL-TK Renilla plasmid as an internal control. For drug-treatment experiments, cells were treated with 1.5 μM FLLL32 or vehicle control after transfection. Luciferase activity was measured using a Double-Luciferase Reporter Assay Kit (GeneCreate, JKR23008-100T) according to the manufacturer’s instructions. Relative luciferase activity was calculated as the ratio of firefly luciferase activity to Renilla luciferase activity and normalized to the corresponding control group.

### Bio-layer interferometry (BLI) assay

Bio-layer interferometry was performed using an Octet RED96e system (ForteBio, Sartorius) to examine the interaction of FLLL32 with STAT3-WT or STAT3-TB2 proteins. Purified proteins were biotinylated using EZ-Link NHS-PEG12-Biotin and immobilized onto streptavidin biosensors. The assay buffer contained 20 mM HEPES, 300 mM NaCl, 10% glycerol, 0.02% Tween-20, and 10% DMSO at pH 7.4. After baseline stabilization, the sensors were exposed to FLLL32 at concentrations ranging from 1.953 to 500 μM for 120 s, followed by dissociation in assay buffer for 120 s. All measurements were performed at 26 °C with orbital shaking at 1000 rpm. DMSO-only controls were used for reference subtraction, and the data were analyzed using Octet Analysis Studio software. FLLL32 bound to STAT3-WT with a KD of 409.5 μM, whereas STAT3-TB2 did not show a clear concentration-dependent binding response.

### Immunohistochemistry and H-score evaluation

For immunohistochemistry (IHC) analysis, xenograft tumor tissues were fixed in formalin, embedded in paraffin, and cut into sections. The sections were deparaffinized, rehydrated through a graded alcohol series, and subjected to antigen retrieval using sodium citrate buffer. Endogenous peroxidase activity was blocked with 3% H₂O₂, and nonspecific binding was blocked with 5% normal goat serum. The sections were then incubated with primary antibodies against Ki67 (Proteintech, 27309-1-AP), Phospho-STAT3 (Tyr705) (CST, 9145), or GPX4 (HUABIO, ET1706-45) at 4 °C overnight. After incubation with HRP-conjugated secondary antibodies, the sections were visualized using DAB substrate and counterstained with hematoxylin. Images were captured using a Nikon microscope.

IHC staining was evaluated using the H-score method. For each tumor, three randomly selected tumor fields were analyzed under the same magnification. Staining intensity was classified as 0, negative; 1 +, weak; 2 +, moderate; and 3 +, strong. The percentage of tumor cells at each staining intensity was recorded, and the H-score was calculated as follows: H-score = 1 × percentage of weakly stained cells +2 × percentage of moderately stained cells +3 × percentage of strongly stained cells. The final H-score for each tumor was calculated as the average value from three fields.

### Mouse tumor xenograft model

The animal procedures were approved by the Institutional Animal Care and Use Committee of West China Hospital, Sichuan University and conducted in accordance with the Guide for the Care and Use of Laboratory Animals. Five-week-old male BALB/c-nu/nu mice weighing 20–23 g were obtained from Nanjing Biomedical Research Institute of Nanjing University (Nanjing, China) and housed under SPF conditions with a 12 h light/12 h dark cycle.

For the subcutaneous xenograft model, HCT116 cells were harvested and resuspended in PBS. A total of 1 × 10^7^ HCT116 cells in 100 μL PBS were subcutaneously injected into the dorsal flank of mice. When tumor volumes reached approximately 100 mm^3^, mice were randomly divided into two groups (*n* = 6 per group). FLLL32 was formulated in vehicle containing 5% DMSO, 40% PEG300, 5% Tween-80, and 50% ddH₂O and administered intraperitoneally at 50 mg/kg once daily. Tumor volume and body weight were measured using a digital caliper every two days for 20 days. Tumor volume was calculated using the formula: V = 0.5 × length × width^2^. At the endpoint, mice were euthanized by CO₂ inhalation, and tumors were collected for further analysis.

### Statistical analysis

All statistical analyses and graphics were performed using GraphPad (v8.0) software. For in vitro experiments, data were displayed as the mean ± SD from three independent experiments unless otherwise stated. For animal experiments, n indicates the number of mice per group. Comparisons between two groups were performed using an unpaired two-tailed Student’s t-test. Comparisons among multiple groups were performed using one-way ANOVA followed by appropriate multiple-comparison tests. Tumor growth curves were analyzed using two-way repeated-measures ANOVA. *p* < 0.05 was considered as statistically significant. *p < 0.05, **p < 0.01, ***p < 0.001, ****p < 0.0001.

## Supplementary Information

Supplementary Material 1.

## Data Availability

All data and materials are available from the corresponding authors upon reasonable request. Raw sequencing data are publicly available in GSA-Human (HRA019190).
