# Supplementary material for: Curcumin analogue FLLL32 induces ferroptosis by targeting the STAT3-GPX4/SLC40A1 axis in colorectal cancer
Source: Mol Biomed. 2026 Oct 20;7:217. doi: 10.1186/s43556-026-00575-5 (PMC13683295; doi:10.1186/s43556-026-00575-5)
Supplement: Supplementary file 1 [file 43556_2026_575_MOESM1_ESM.pdf]

Supplementary Materials for

Curcumin analogue FLLL32 induces ferroptosis by targeting the STAT3-GPX4/SLC40A1 axis in colorectal cancer

Tong Wu[1#], Mengqiu Hao[1#], Qiuye Luo[1], Sicheng Liu[1], Yaguang Zhang[1], Yang Zhang[2], Xuyang Yang[2*], Junhong Han[1*]

*** Correspondence to**
Xuyang Yang, E-mail: yangxuyang@wchscu.cn
Junhong Han, E-mail: hjunhong@scu.edu.cn

This supplementary file includes:

Supplemental Materials and Methods
Fig. S1-S6
Table S1-S2

Supplemental Materials and Methods

Cell viability and colony formation assays

For cell viability assays, a total of 8000 cells were seeded in 96-well plates, and Cell Counting Kit-8 (CCK-8, TargetMol, C0005) was added and incubated for 1 h after treatment. The absorbance was measured at 450 nm with a microplate reader (BioTek).

For colony formation assays, cells were seeded into 48-well plates at 1000 cells per well. Cells were treated with the indicated drugs for 24 h, followed by replacement with fresh medium. Cells were cultured for 1 week, fixed with 4% paraformaldehyde (PFA) for 30 min and stained with 0.1% crystal violet for 20 min at room temperature. Colonies were imaged and quantified.

Wound-healing assay

HCT116 cells were seeded into 6-well plates and cultured overnight until they reached more than 95% confluence. A linear wound was generated in each well using a sterile pipette tip. Detached cells were removed by washing with PBS two to three times. Cells were then cultured in medium containing 2% FBS and treated with DMSO or FLLL32. The use of serum-reduced medium was intended to minimize the contribution of cell proliferation to wound closure. Images of the same wound areas were captured at 0 and 48 h using an inverted microscope. The wound healing distance ratio was quantified using ImageJ software and calculated as follows: wound healing distance ratio = wound width at 48 h / wound width at 0 h.

EdU incorporation assay

EdU was detected using a BeyoClick™ EdU Cell Proliferation Kit with Alexa Fluor 488 (Beyotime, C0085L) according to the manufacturer's protocol. Cells were seeded on coverslips in 24-well plates and treated as indicated. Cells were incubated with 50 μM EdU for 2 h, fixed with 4% paraformaldehyde, permeabilized with 0.5% Triton X-100, and incubated with the reaction solution according to the manufacturer's protocol. Nuclei were stained with Hoechst 33342. Images were captured using a fluorescence microscope (Nikon).

Transmission electron microscopy

HCT116 cells were seeded in 10-cm dishes and treated with DMSO or FLLL32 for 48 h. After treatment, cells were washed with PBS, harvested by trypsinization, and collected by centrifugation. The cell pellets were gently washed with PBS and fixed with 3% glutaraldehyde in 0.1 M PBS at 4 °C. After primary fixation, samples were post-fixed with 1% osmium tetroxide, dehydrated through a graded ethanol series, embedded in epoxy resin, and cut into ultrathin sections. Sections were stained with uranyl acetate and lead citrate and examined using a transmission electron microscope. Mitochondrial morphology was evaluated based on mitochondrial size, membrane density, and cristae structure. Mitochondria showing shrinkage, increased membrane density, or reduced/absent cristae were considered damaged.

Nuclear-cytoplasmic fractionation

Nuclear and cytoplasmic proteins were extracted using a nuclear and cytoplasmic protein extraction kit (Beyotime P0027) according to the manufacturer's instructions. Briefly, cells were harvested after treatment and separated into cytoplasmic and nuclear fractions. Equal amounts of protein from each fraction were analyzed by Western blot. Lamin B1 and β-Tubulin were used as nuclear and cytoplasmic

markers.

Plasmid constructions, gene knockdown and overexpression

Gene knockdown was performed using shRNA-based lentiviral vectors. STAT3 was knocked down using shSTAT3-1 (3′UTR), with target sequences listed in Table S2. FTH1 and FTL knockdown were achieved using pLVX-U6-FTH1-shRNA1-EGFP-E2A-Puro (MiaoLing, P23659) and pLV3-U6-FTL-shRNA1-mCherry-Blast (MiaoLing, G106453), respectively. For overexpression experiments, pcDNA3.1(+)-3×HA-GPX4-Neo (PPL, PPL01669-2d) and pLV3-CMV-SLC40A1-3×FLAG-Puro (MiaoLing, P95542) were used. Scramble shRNA and pLV3-EV empty vectors were used as negative controls.

For STAT3 rescue and mutant experiments, human STAT3 was amplified from HCT116 cDNA and cloned into the pLVX-C-3HA vector using the ClonExpress II One Step Cloning Kit (Vazyme, C112-01). The STAT3-TB2 mutant carrying Glu638Ala and Met726Ala substitutions was generated by site-directed mutagenesis PCR and verified by DNA sequencing. Lentiviruses were produced in HEK293T cells by co-transfecting the indicated plasmids with psPAX2 and pMD2.G. Viral supernatants were collected 24-72 h after transfection, filtered through a 0.45 μm filter, and used to transduce HCT116 and HT29 cells. Cells were selected with puromycin at 2 μg/mL for 48-72 h when applicable. Knockdown and overexpression efficiencies were confirmed by RT-qPCR and/or Western blotting. The shRNA target sequences and mutagenesis primers are listed in Table S2.

Molecular docking

Molecular docking was performed to predict the potential binding mode between FLLL32 and STAT3. The three-dimensional structure of FLLL32 was obtained from the PubChem database (PubChem CID: 68019246). The predicted structure of human STAT3 was obtained from the AlphaFold Protein Structure Database (AlphaFold ID: A0A7I2YQR5_HUMAN). Protein and ligand structures were prepared and converted to PDBQT format before docking. Docking analysis was performed using AutoDock Vina 1.2.2. The docking grid was set to cover the predicted binding region of STAT3 containing residues around Glu638 and Met726. Docking poses were ranked according to the predicted binding affinity, and the top-ranked conformation was selected for subsequent analysis. The interaction between FLLL32 and STAT3 was visualized using PyMOL and/or Discovery Studio. Amino acid residues located within 3 Å of FLLL32 were analyzed as potential interacting residues. Based on the docking results, Glu638 and Met726 were selected for subsequent mutagenesis analysis.

Recombinant STAT3 protein expression and purification

The coding sequence of human STAT3 was cloned into the pET-28a vector containing an N-terminal 6×His tag. The STAT3-TB2 mutant was generated by alanine substitution of Glu638 and Met726 near the predicted FLLL32-binding region. The recombinant plasmids were transformed into BL21 (DE3) Escherichia coli. Protein expression was induced with 0.5 mM IPTG at 16 °C overnight. Bacterial pellets were resuspended in buffer A containing 25 mM Tris-HCl, 300 mM NaCl, and 5% glycerol, and lysed by homogenization. Soluble proteins were purified using Ni-NTA affinity chromatography followed by size-exclusion chromatography. Purified STAT3-WT and STAT3-TB2 proteins were used for subsequent BLI analysis.

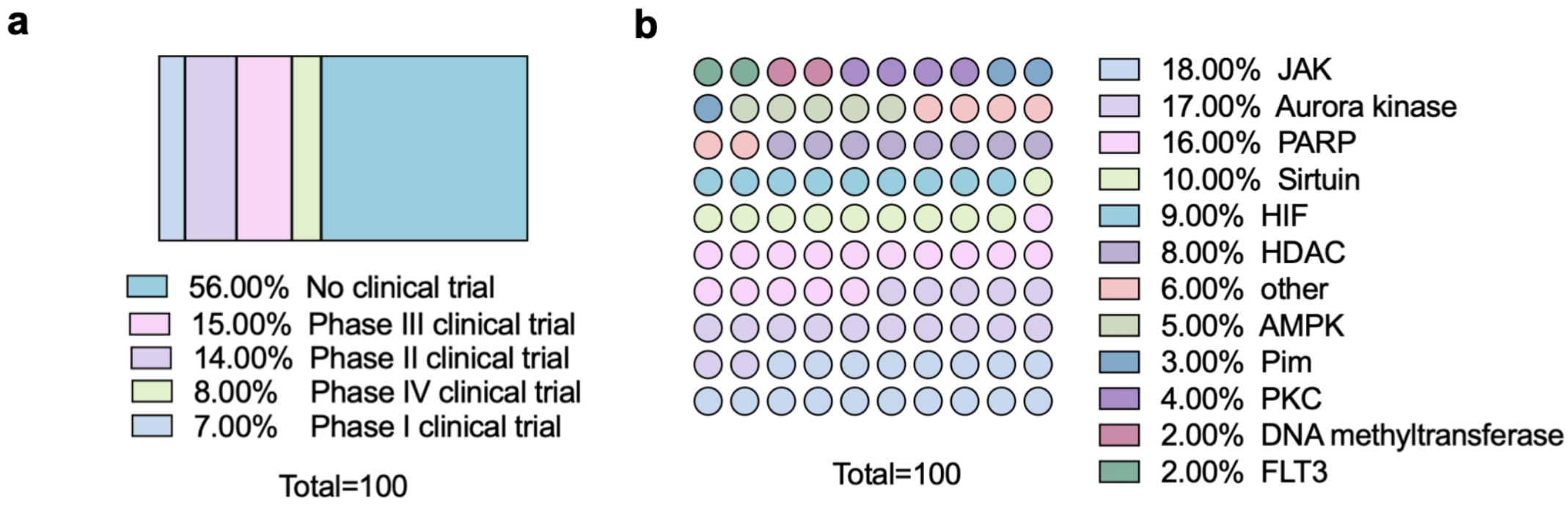

Fig. S1 Composition of the screening library.

a. Distribution of compounds in the screening library according to clinical development stage. **b.** Distribution of compounds according to target category.

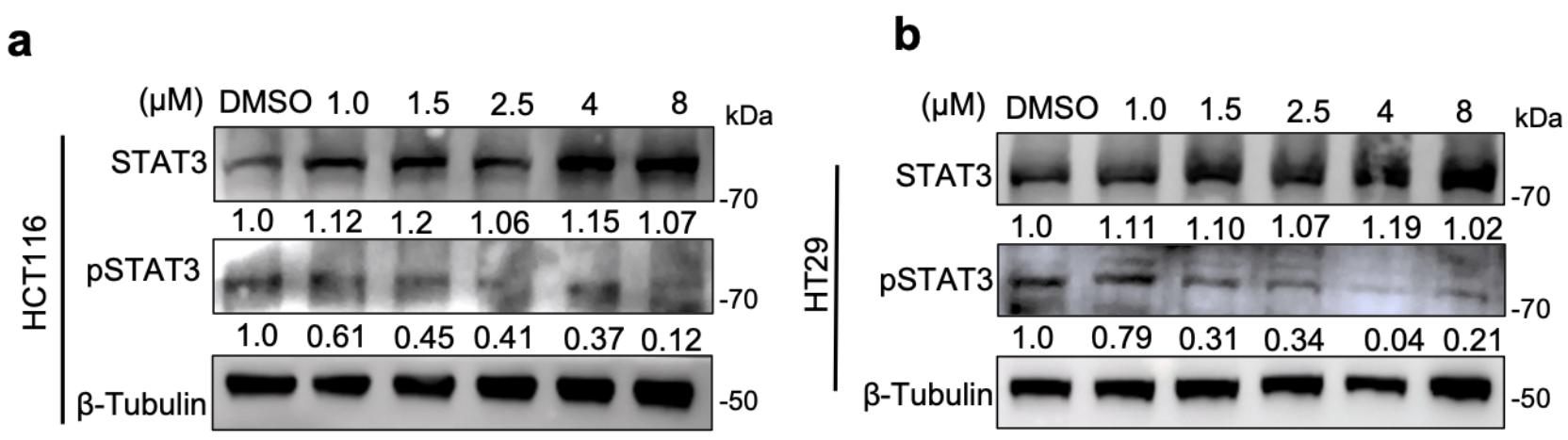

Fig. S2 FLLL32 suppresses pSTAT3 in a dose-dependent manner.

a-b. Western blot analysis of STAT3 and pSTAT3 in HCT116 and HT29 cells treated with the indicated concentrations of FLLL32. β-Tubulin was used as the loading control.

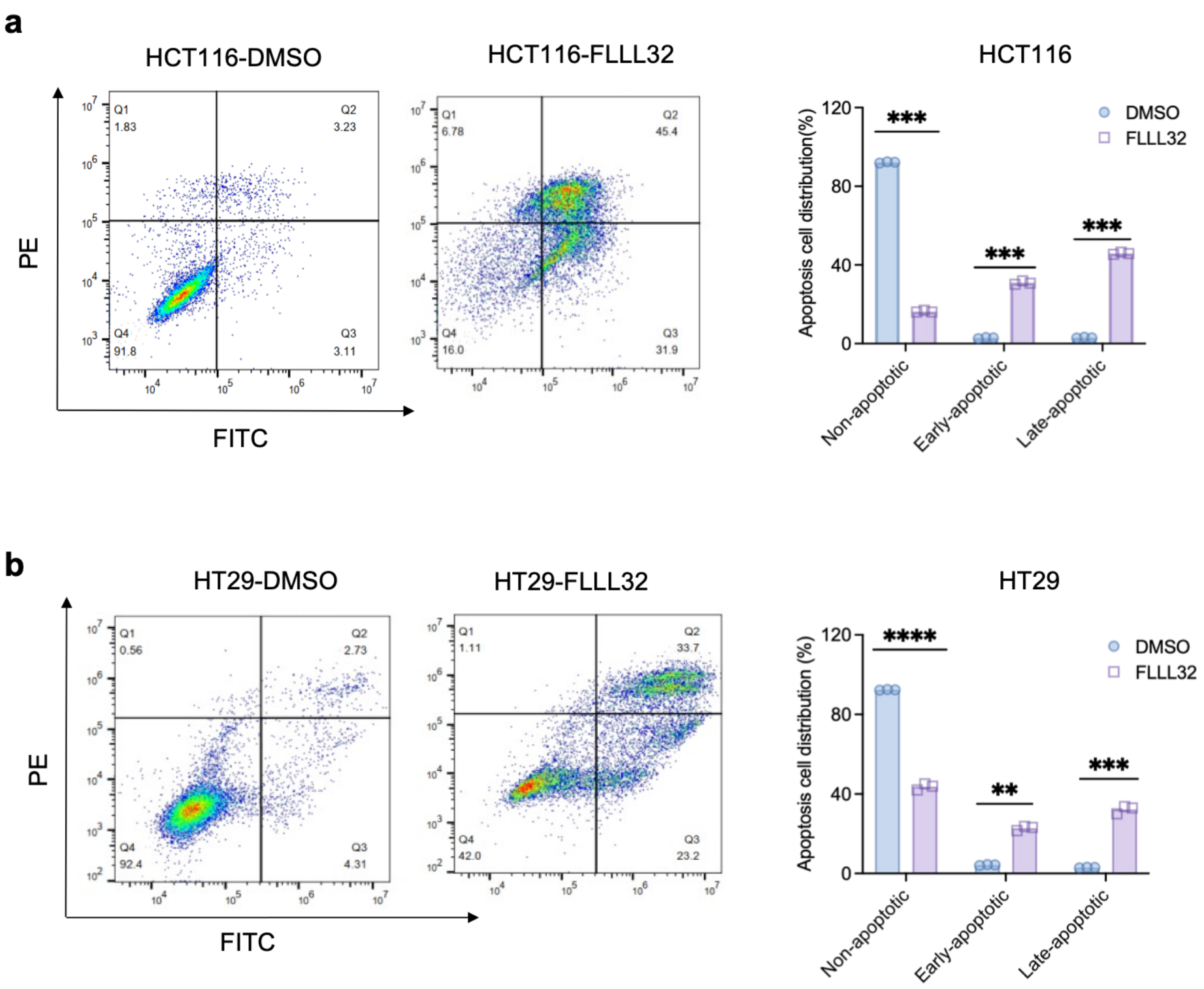

Fig. S3 FLLL32 induces apoptotic features in CRC cells.

a-b. Annexin V-FITC/PI flow cytometry plots and quantification of non-apoptotic, early apoptotic, and late apoptotic cell populations in HCT116 and HT29 cells. Data are presented as the mean ± SD, n = 3, $^{**}p < 0.01$, $^{***}p < 0.001$, $^{****}p < 0.0001$.

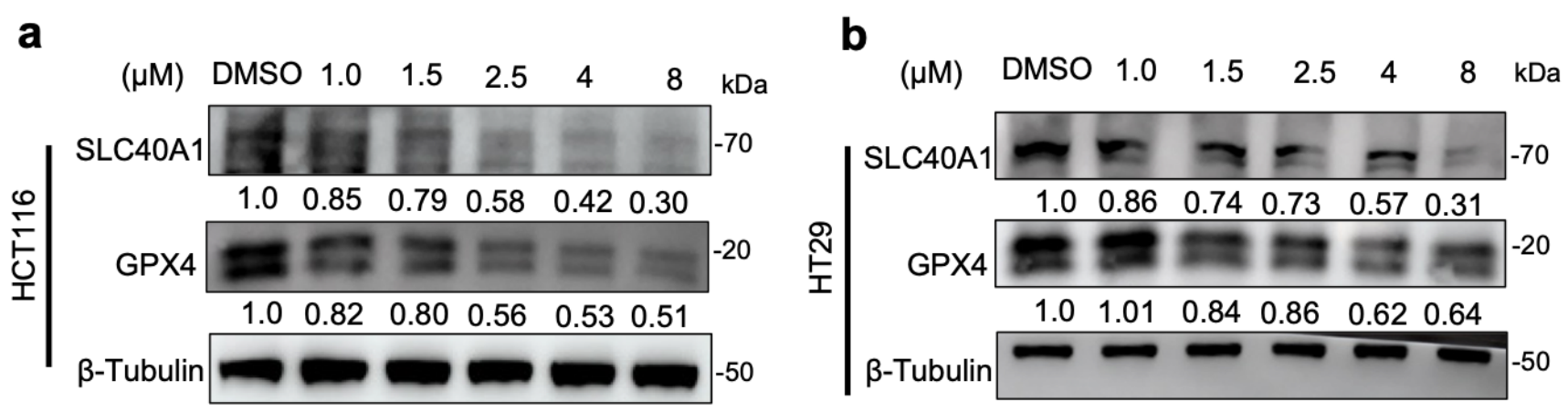

Fig. S4 FLLL32 suppresses GPX4 and SLC40A1 in a dose-dependent manner.

a-b. Western blot analysis of GPX4 and SLC40A1 in HCT116 and HT29 cells treated with the indicated concentrations of FLLL32. β-Tubulin was used as the loading control.

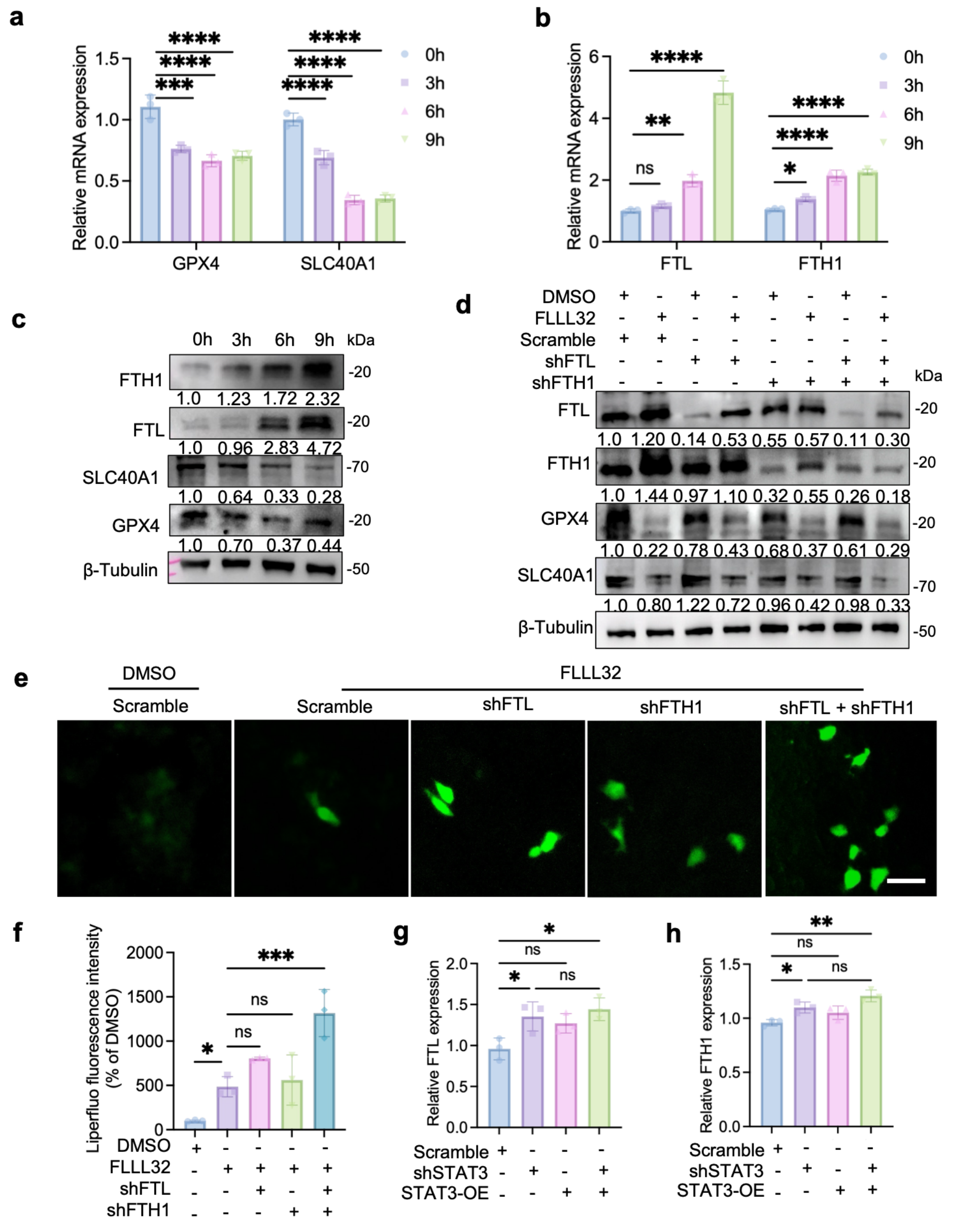

Fig. S5 FTH1 and FTL act as compensatory regulators of ferroptosis-associated lipid peroxidation. a. qRT-PCR showing GPX4 and SLC40A1 expression in HCT116 cells treated with FLLL32 for the indicated times. **b.** qRT-PCR showing FTH1 and FTL expression in HCT116 cells treated with FLLL32 for the indicated times. **c.** Western blot showing FTH1, FTL, SLC40A1, and GPX4 expression in HCT116 cells treated with FLLL32 for the indicated times. β-Tubulin was used as the loading control. **d.** Western blot analysis of FTL, FTH1, GPX4, and SLC40A1 in HCT116 cells with FTL and/or FTH1 knockdown after DMSO or FLLL32 treatment. β-Tubulin served as the loading control. **e.** Representative Liperfluo staining images of HCT116 cells with FTL and/or FTH1 knockdown after FLLL32 treatment. Scale bar, 50 μm. **f.** Quantification of Liperfluo fluorescence intensity in HCT116 cells with FTL and/or FTH1 knockdown after FLLL32 treatment. **g-h.** qRT-PCR analysis of FTL (g) and FTH1 (h) mRNA expression in HCT116 cells after STAT3 knockdown, STAT3 overexpression, or STAT3 re-expression in STAT3-knockdown cells. Data are presented as the mean ± SD, n = 3. *$p < 0.05$, **$p < 0.01$, ***p <

0.001, ****p < 0.0001.

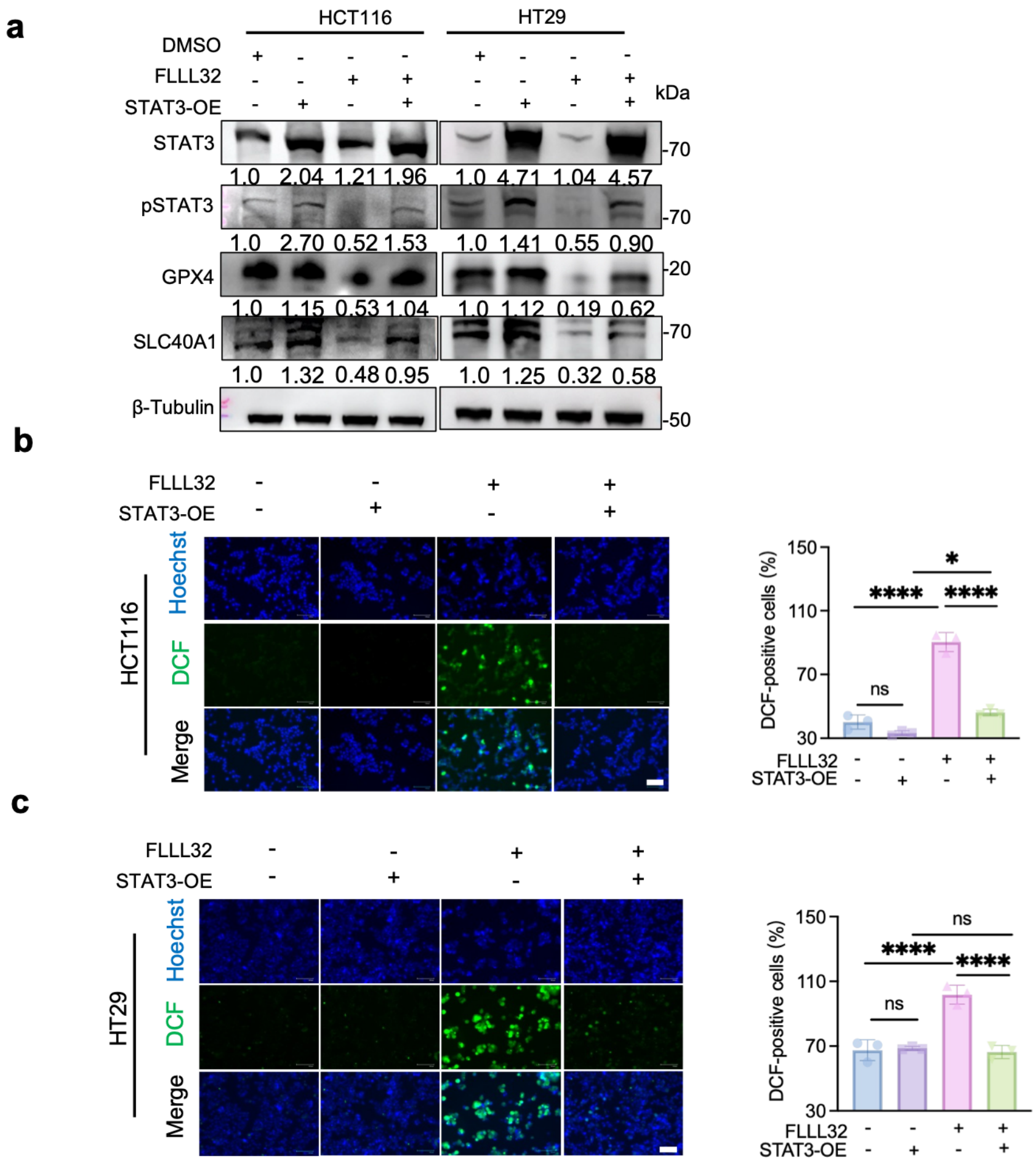

Fig. S6 STAT3 modulates GPX4/SLC40A1 expression in response to FLLL32.

a. Western blot analysis of STAT3, pSTAT3, GPX4, and SLC40A1 in HCT116 and HT29 cells with or without STAT3 overexpression after DMSO or FLLL32 treatment. β-Tubulin served as the loading control. **b-c.** Representative DCF staining images and quantification of DCF-positive cells in HCT116 (b) and HT29 (c) cells with or without STAT3 overexpression after FLLL32 treatment. Hoechst was used for nuclear staining. Scale bar, 100 μm. Data are presented as the mean ± SD, n = 3. *p < 0.05, ****p < 0.0001.

Table S1

Catalog No.	Drug name	Target pathway	Inhibition rate
S1085	Belinostat (PXD101)	HDAC	1
S7259	FLLL32	JAK	0.98444444
S1249	JNJ-7706621	Aurora Kinase,CDK	0.954545455
S1030	Panobinostat (LBH589)	HDAC	0.933333333
S1515	Pracinostat (SB939)	HDAC	0.875
S2018	ENMD-2076 L-(+)-Tartaric acid	Aurora Kinase,FLT3,VEGFR	0.8
S2736	Fedratinib (SAR302503, TG101348)	JAK	0.666666667
S2170	Givinostat (ITF2357)	HDAC	0.642857143
S2214	AZ 960	JAK	0.64
S2162	AZD1480	JAK	0.636363636
S7104	AZD1208	Pim	0.633333333
S2391	Quercetin	Src,Sirtuin,PKC,PI3K	0.586206897
S2219	Momelotinib (CYT387)	JAK	0.5625
S1529	Hesperadin	Aurora Kinase	0.52
S1154	SNS-314 Mesylate	Aurora Kinase	0.45
S1098	Rucaparib (AG-014699,PF-01367338) phosphate	PARP	0.45
S1053	Entinostat (MS-275)	HDAC	0.44
S2796	WP1066	JAK	0.409090909
S1134	AT9283	Aurora Kinase,Bcr-Abl,JAK	0.421052632
S1233	2-Methoxyestradiol (2-MeOE2)	HIF	0.368421053
S2178	AG-14361	PARP	0.363636364
S1048	Tozasertib (VX-680, MK-0457)	Aurora Kinase	0.3125
S8138	Molidustat (BAY 85-3934)	HIF	0.296296296
S2740	GSK1070916	Aurora Kinase	0.291666667
S7238	NVP-TNKS656	PARP	0.277777778
S1107	Danusertib (PHA-739358)	Aurora Kinase,Bcr-Abl,c-RET,FGFR	0.272727273
S1181	ENMD-2076	Aurora Kinase,FLT3,VEGFR	0.25
S7946	KC7F2	HIF	0.25
S1133	Alisertib (MLN8237)	Aurora Kinase	0.210526316
S7612	PX-478 2HCl	HIF	0.210526316
S7541	Decernotinib (VX-509)	JAK	0.208333333
S2542	Phenformin HCl	AMPK	0.206896552
S1004	Veliparib (ABT-888)	PARP	0.19047619
S2902	S-Ruxolitinib (INCB018424)	JAK	0.133333333
S2298	Fisetin	Sirtuin	0.130434783
S8057	Pacritinib (SB1518)	FLT3,JAK	0.1
S1422	Droxinostat	HDAC	0.095238095
S2554	Daphnetin	PKA,EGFR,PKC	0.08
S7634	Cerdulatinib (PRT062070,	JAK	0.0625

	PRT2070)		
S2791	Sotrastaurin	PKC	0.058823529
S7318	HTH-01-015	AMPK	0.05
S1703	Divalproex Sodium	HDAC	0.038461538
S2718	TAK-901	Aurora Kinase	0
S1007	Roxadustat (FG-4592)	HIF	0
S1087	Iniparib (BSI-201)	PARP	0
S1168	Valproic acid sodium salt (Sodium valproate)	GABA Receptor,HDAC,Autophagy	0
S2158	KW-2449	Aurora Kinase,Bcr-Abl,FLT3	0
S2719	AMG-900	Aurora Kinase	0
S2804	Sirtinol	Sirtuin	0
S2919	IOX2	HIF	0
S1129	SRT1720 HCl	Sirtuin	0
S1378	Ruxolitinib (INCB018424)	JAK	0
S2806	CEP-33779	JAK	0
S1132	INO-1001 (3-Aminobenzamide)	PARP	0
S1541	Selisistat (EX 527)	Sirtuin	0
S2179	Gandotinib (LY2784544)	JAK	0
S2686	NVP-BSK805 2HCl	JAK	0
S2770	MK-5108 (VX-689)	Aurora Kinase	0
S2851	Baricitinib (LY3009104, INCB028050)	JAK	0
S4246	Tranylcypromine (2-PCPA) HCl	MAO	0
S1100	MLN8054	Aurora Kinase	0
S1451	Aurora A Inhibitor I	Aurora Kinase	0
S2198	SGI-1776 free base	Pim	0
S4294	Procainamide HCl	DNA Methyltransferase,Sodium Channel	0
S1060	Olaparib (AZD2281, Ku-0059436)	PARP	0
S2886	PJ34	PARP	0
S7029	AZD2461	PARP	0
S7300	PJ34 HCl	PARP	0
S7438	ME0328	PARP	0
S8005	SMI-4a	Pim	0
S7625	Niraparib (MK-4827) tosylate	PARP	0
S7605	Filgotinib (GLPG0634)	JAK	0
S8038	UPF 1069	PARP	0
S1055	Enzastaurin (LY317615)	PKC	0
S2250	(-)-Epigallocatechin Gallate	DNA Methyltransferase,HER2,Telomerase,EGFR,Fatty Acid Synthase	0

S8370	BGP-15 2HCl	PARP	0
S7144	BMS-911543	JAK	0
S7617	Tasquinimod	HDAC	0
S8270	SRT2183	Sirtuin	0
S4710	Picolinamide	PARP	0
S8419	E7449	PARP	0
S2407	Curcumol	JAK	0
S7207	Bisindolylmaleimide IX (Ro 31-8220 Mesylate)	PKC	0
S7577	AGK2	Sirtuin	0
S7650	Peficitinib (ASP015K, JNJ-54781532)	JAK	0
S7953	ETC-1002	AMPK,LDL	0
S4735	Salvianolic acid B	Sirtuin	0
S7958	Lificiguat(YC-1)	HIF	0
S8171	Daprodustat (GSK1278863)	HIF	0
S8441	LW 6	HIF	0
S1899	Nicotinamide (Vitamin B3)	Sirtuin	0
S7065	MK-8745	Aurora Kinase	0
S7845	SirReal2	Sirtuin	0
S8245	Thiomyristoyl	Sirtuin	0
S2911	Go 6983	PKC	0
S7119	Go6976	FLT3,JAK,PKC	0
S7317	WZ4003	AMPK	0
S8249	HPI-4 (Ciliobrevin A)	Hedgehog/Smoothened	0
S1950	Metformin HCl	Autophagy	0
S1802	AICAR (Acadesine)	AMPK	-0.111111111

Table S2 Primers

Name	Sequence (5′-3′)
shSTAT3-1(UTR)-F	CCGGCATCTCAAACTACTAACTTTGCTCGAGCAAAGTTAGTAGTTTGAGATGTTTTTG
shSTAT3-1(UTR)-R	AATTCAAAAACATCTCAAACTACTAACTTTGCTCGAGCAAAGTTAGTAGTTTGAGATG
STAT3-Glu638-A-F	CGTGGGACCATACACAAAGCAGCAGCTGAACA
STAT3-Glu638-A-R	TTGTGTATGGTCCCACGGACTGGATCTGGGTCTT
STAT3-Met726-A-F	ATTGACCTGCCGGGGTCCCCCCGCACTTTAGATTC
STAT3-Met726-A-R	GGGACCCCGGCAGGTCAATGGTATTGCTGCAGG
GPX4-ChIP-F	GCTGTCAACCAGCCGGATA
GPX4-ChIP-R	GGACTTGTTGTTGGCGACTG
SLC40A1-ChIP-F	TTCCAGCACCTGACGCTTAG
SLC40A1-ChIP-R	GTTTTCCTGCTCTCCGGGAA
H-qp-SLC40A1-F	GAGACAAGTCCTGAATCTGTGCC
H-qp-SLC40A1-R	TTCTTGCAGCAACTGTGTCACAG
H-qp-FTL-F	TACGAGCGTCTCCTGAAGATGC
H-qp-FTL-R	GGTTCAGCTTTTTCTCCAGGGC
H-qp-FTH1-F	TGAAGCTGCAGAACCAACGAGG
H-qp-FTH1-R	GCACACTCCATTGCATTCAGCC
H-qp-GPX4-F	ACAAGAACGGCTGCGTGGTGAA
H-qp-GPX4-R	GCCACACACTTGTGGAGCTAGA
Hqpcr-GAPDH-F	GGAGCGAGATCCCTCCAAAAT
Hqpcr-GAPDH-R	GGCTGTTGTCATACTTCTCATGG
SLC40A1-WT-promoter reporter insert	GGAATCCCCACCCACCAAGCTCGCGCTGGAGCTTTGCACTGCGACCGTCCGGCGGCCGCCCTTTCCCTGAACTGCGGGGTAGCCGGTGCGCAGCGCTTTCTTCCAGCACCTGACGCTTAGTTTCGCGCAGAATCTCCCTACGCCGCCCCGCCGGCTCCACGCGCCTTCCTCCTTTTCCCAGCCCCACGGCCG
SLC40A1-MT-promoter reporter insert	GGAATCCCCACCCACCAAGCTCGCGCTGGAGCTTTGCACTGCGACCGTCCGGCGGCCGCCCGGCTTTCAGCATGCGGGGTAGCCGGTGCGCAGCGCTTTCTTCCAGCACCTGACGCTTAGTTTCGCGCAGAATCTCCCTACGCCGCCCCGCCGGCTCCACGCGCCTTCCTCCTTTTCCCAGCCCCACGGCCG
GPX4-WT-promoter reporter insert	GGCAAATCCCGGCCGAGCCTTTGGCTCACGCATGTGATCCCAGCACTTTTGGGAAGCTGAGGCTGGAGGATCACTTGAGGTCAGAAGTTTGAGACCAGCCTAGGCAACATAGCGAGACCCCCATGTCTACAAAACAATAATTAGCCAGGAATGGTGGATGAGCCTGTTGTC
GPX4-MT-promoter reporter insert	GGCAAATCCCGGCCGAGCCTTTGGCTCACGCATGTGATCCCAGCAACTCACGTAGCCTGAGGCTGGAGGATCACTTGAGGTCAGAAGTTTGAGACCAGCCTAGGCAACATAGCGAGACCCCCATGTCTACAAAACAATAATTAGCCAGGAATGGTGGATGAGCCTGTTGTC
